# Quantifying the Human Subchondral Trabecular Bone Microstructure in Osteoarthritis with Clinical CT

**DOI:** 10.1002/advs.202201692

**Published:** 2022-06-07

**Authors:** Tamás Oláh, Xiaoyu Cai, Liang Gao, Frédéric Walter, Dietrich Pape, Magali Cucchiarini, Henning Madry

**Affiliations:** ^1^ Center of Experimental Orthopaedics Saarland University Kirrberger Straße 100, Building 37 Homburg Saar D‐66421 Germany; ^2^ Cartilage Net of the Greater Region Kirrberger Straße 100, Building 37 Homburg Saar D‐66421 Germany; ^3^ Clinique d'Eich Centre Hospitalier de Luxembourg 78 Rue d'Eich Luxembourg 1460 Luxembourg

**Keywords:** 3D microstructure, computed tomography, microcomputed tomography, multislice computed tomography, osteoarthritis of knee, subchondral bone

## Abstract

Osteoarthritis (OA) is characterized by critical alterations of the subchondral bone microstructure, besides the well‐known cartilaginous changes. Clinical computed tomography (CT) detection of quantitative 3D microstructural subchondral bone parameters is applied to monitor changes of subchondral bone structure in different stages of human OA and is compared with micro‐CT, the gold standard. Determination by clinical CT (287 µm resolution) of key microstructural parameters in tibial plateaus with mild‐to‐moderate and severe OA reveals strong correlations to micro‐CT (35 µm), high inter‐ and intraobserver reliability, and small relative differences. In vivo, normal, mild‐to‐moderate, and severe OA are compared with clinical CT (331 µm). All approaches detect characteristic expanded trabecular structure in severe OA and fundamental microstructural correlations with clinical OA stage. Multivariate analyses at various in vivo and ex vivo imaging resolutions always reliably separate mild‐to‐moderate from severe OA (except mild‐to‐moderate OA from normal), revealing a striking similarity between 287 µm clinical and 35 µm micro‐CT. Thus, accurate structural measurements using clinical CT with a resolution near the trabecular dimensions are possible. Clinical CT offers an opportunity to quantitatively monitor subchondral bone microstructure in clinical and experimental settings as an advanced tool of investigating OA and other diseases affecting bone architecture.

## Graphical abstract

Critical subchondral bone microstructural alterations characteristic to osteoarthritis (OA) are not yet utilized in clinical diagnostics. Based on such changes, clinical computed tomography (CT) reliably differentiates between mild‐to‐moderate and severe OA, similarly to the gold standard micro‐CT. Clinical CT detection of microstructural parameters may advance the assessment of OA and other diseases affecting bone architecture in research and therapy.

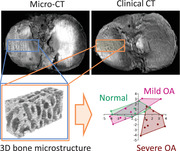

## 1 Introduction

Osteoarthritis (OA), the most common debilitating joint disorder worldwide, is characterized by subchondral bone alterations besides articular cartilage degeneration.^[^ ^1^ ^]^ Changes of its microstructure include subchondral bone plate sclerosis and degradation of subarticular trabeculae (i.e., the subarticular spongiosa), followed by an increase of their volume and structural complexity.^[^ ^2^ ^]^ However, this multifaceted pattern of 3D changes largely escapes detection on plain radiographs,^[^ ^2d^ ^]^ and indices of bone texture, although they may correlate with certain true 3D bone structural parameters,^[^ ^3^ ^]^ have limited clinical and anatomical interpretability.^[^ ^4^ ^]^ Radiographic categories based on the Kellgren–Lawrence (KL) scoring system such as joint space narrowing are the basis of clinically diagnosing OA.^[^ ^2^, ^5^ ^]^ Applicability of magnetic resonance imaging (MRI), the most important imaging modality in a research context,^[^ ^6^ ^]^ to quantitate 3D bone structure^[^ ^7^ ^]^ is reduced as specific sequences are needed and long^[^ ^8^ ^]^ scan durations may cause motion artifacts.^[^ ^9^ ^]^ Computed‐tomography (CT), used to examine bony parts, offers a superior visualization of mineralized tissues,^[^ ^10^ ^]^ and may be used to evaluate bone mineral density.^[^ ^11^ ^]^ If enhanced by intraarticular (i.a.) injection of a contrast medium, such arthro‐CT is considered the imaging reference standard for in vivo assessment of cartilage thickness,^[^ ^6^ ^]^ and thus frequently used for a detailed qualitative assessment of cartilage damage, cysts, and osteophytes in 3D.^[^ ^12^ ^]^ Quantitative clinical CT‐based evaluation of the human subchondral bone microstructure has not been forthcoming so far, possibly because of reduced spatial resolution (90–250 µm, near trabecular dimensions) and challenging technical issues such as segmentation and volume of interest (VOI) positioning.^[^ ^10^ ^]^ Micro‐CT allows to nondestructively visualize the microstructure of bone, with an ≈1 to >100 µm^[^ ^13^ ^]^ isotropic resolution, and to quantify structural parameters with a high degree of accuracy. The extremely detailed structural information provides an unparalleled insight into the true 3D nature of trabecular morphology,^[^ ^14^ ^]^ bone remodeling,^[^ ^15^ ^]^ and biological processes.^[^ ^16^ ^]^ Micro‐CT represents the reference standard for evaluating 3D bone microstructure of small (few cubic centimeters) probes, but is, at present, not applicable to in vivo human use.^[^ ^17^ ^]^

The aim of this study was to provide evidence that clinical CT may be used to monitor microstructural bone changes in OA, possibly applicable clinical investigative studies. We first hypothesized that clinical CT is capable of quantifying the altered subchondral trabecular microstructure in human knee OA^[^ ^2^ ^]^ in a precision similar to high‐resolution micro‐CT. Second, we hypothesized that the measured 3D structural indices show high intra‐ and interobserver reliability and strong correlations between clinical CT and micro‐CT. Third, we hypothesized that mild‐to‐moderate and severe OA can reliably be differentiated by multivariate analyses based on micro‐CT and clinical CT datasets of various resolutions.

## 2 Results

### 2.1 Clinical CT Accurately Measures Noncalcified Cartilage and Calcified Tissue Thickness Differences between Mild‐to‐Moderate and Severe OA

**Figure 1:**
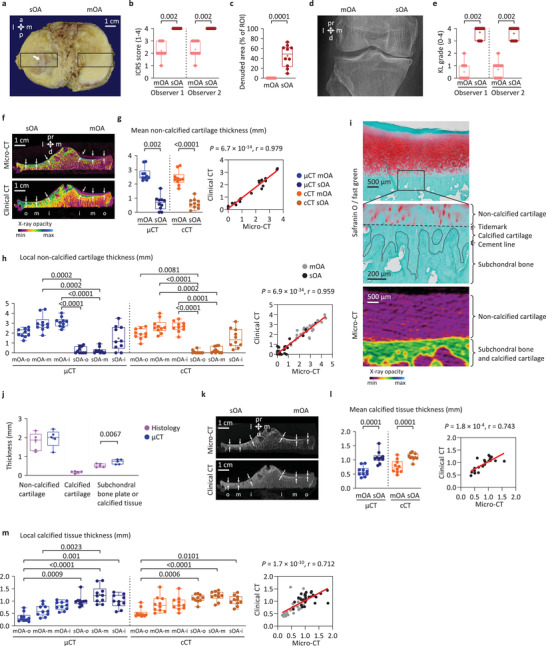
Manual measurement of noncalcified cartilage and calcified tissue thickness. a) Regions of interests (ROIs) in the medial and lateral tibial plateaus (arrow: denudation). b) Classification of the ROIs based on ICRS scores^[^ ^19^ ^]^ into severe OA (sOA; score 4, cartilage erosion penetrating into the subchondral bone) or mild‐to‐moderate OA groups (mOA; score ≤ 3, cartilage damage not reaching the subchondral bone) (dots: individual data points, +: mean, whiskers: minimum and maximum, borders: 75th and 25th percentiles). c) Extent of denuded areas. d) Representative X‐ray image and e) KL grades.^[^ ^20^ ^]^ f) Color‐coded CT images showing the locations of noncalcified cartilage thickness measurements. g) Mean noncalcified cartilage thickness of mOA and sOA tibial plateaus, and scatter plot and linear regression showing the correlation between clinical CT and micro‐CT measurements. h) Regional analysis of noncalcified cartilage thickness. i) Safranin O/fast green stained histological section and color‐coded 2D micro‐CT image showing overlapping calcified cartilage and subchondral bone plate. j) Thickness comparison between histology and micro‐CT (*n* = 5). k) CT images showing the locations of calcified tissue thickness measurements. l) Mean calcified tissue thickness of mOA and sOA tibial plateaus, and scatter plot and linear regression showing the correlation between clinical CT and micro‐CT measurements. m) Regional analysis of calcified tissue thickness. *n* = 10 per group. Abbreviations: a, anterior; d, distal; l, lateral; m, medial; p, posterior; pr, proximal, cCT, clinical CT; *μ*CT, micro‐CT; o, outer; m, middle; i, inner subregions. Paired *T*‐test or Wilcoxon test, or Kruskal–Wallis ANOVA.

To examine the performance of clinical CT to distinguish different stages of OA, we selected tibial plateaus as a clinically relevant location similarly affected by OA than distal femora, allowing reproducible VOI positioning^[^ ^18^ ^]^ due to precise anatomical landmarks for reliable analyses. We first classified human tibial plateaus as mild‐to‐moderate or severe OA based on their macroscopic International Cartilage Regeneration and Joint Preservation Society (ICRS) scores (**Figure** **1**a–c) and confirmed with the KL grades (Figure 1d,e). In severe OA, noncalcified cartilage thickness was significantly lower, and calcified tissue (including subchondral bone plate and calcified cartilage) thickness was significantly larger (Figure 1f–m) compared with mild‐to‐moderate OA (all *p* ≤ 0.002) when evaluated with either ex vivo clinical or micro‐CT, while the subregional patterns of changes were similar (Figure 1h,m). CT did not visualize calcified cartilage (Figure 1i,j). Clinical CT measurements showed significant, strong, positive correlations with micro‐CT (noncalcified cartilage thickness: *p* = 6.7 × 10^−14^, *r* = 0.979; calcified tissue thickness: *p* = 1.8 × 10^−4^, *r* = 0.743) (Figure 1g,l).

### 2.2 Clinical CT Reliably Detects Trabecular Structural Differences between Mild‐to‐Moderate and Severe OA with Strong Correlations to Micro‐CT

**Figure 2:**
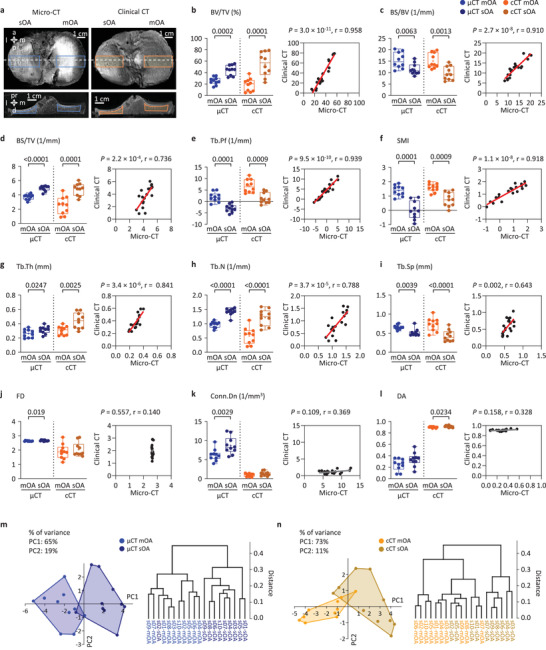
Comparison of the subarticular spongiosa microstructure of mild‐to‐moderate and severe OA tibial plateaus with clinical CT and micro‐CT. a) Position of the evaluated ROIs within the subarticular spongiosa (dashed line: location of the 2D section). Box plots of the measured values, and scatter plot and linear regression showing the correlation between clinical CT and micro‐CT measurements of the b) percent bone volume (BV/TV), c) bone surface‐to‐volume ratio (BS/BV), d) bone surface density (BS/TV), e) trabecular pattern factor (Tb.Pf), f) structure model index (SMI), g) trabecular thickness (Tb.Th), h) trabecular number (Tb.N), i) trabecular separation (Tb.Sp), j) fractal dimension (FD), k) connectivity density (Conn.Dn), and l) degree of anisotropy (DA). *n* = 10 per group; paired *T*‐test or Wilcoxon test. PCA and cluster analysis based on the above subarticular spongiosa parameters determined with m) micro‐CT or n) clinical CT. Data points represent individual mild‐to‐moderate or severe OA tibial plateaus (*n* = 10 per group). Information content (% of variance) of axes for principal components 1 (PC1) and 2 (PC2) are shown above graphs. Abbreviations: a, anterior; d, distal; l, lateral; m, medial; p, posterior; pr, proximal, mOA, mild‐to‐moderate OA; sOA, severe OA; cCT, clinical CT; *μ*CT, micro‐CT.

With both clinical (ex vivo) and micro‐CT methods, values for percent bone volume (BV/TV), bone surface density (BS/TV), trabecular thickness (Tb.Th), and trabecular number (Tb.N) of the subarticular spongiosa in severe OA tibial plateaus were significantly higher (all *p* ≤ 0.0247) than in mild‐to‐moderate OA (**Figure** **2**a–i). Additionally, the bone surface‐to‐volume ratio (BS/BV), trabecular pattern factor (Tb.Pf), structure model index (SMI), and trabecular separation (Tb.Sp) were significantly lower (all *p* ≤ 0.0063) in severe OA (Figure 2a–i). These micro‐CT and clinical CT data showed strong to very strong^[^ ^21^ ^]^ (*r* = 0.643 to 0.958), significant (*p* = 0.002 to 3.0 × 10^−11^), positive Pearson correlations. Fractal dimension (FD), connectivity density (Conn.Dn), and degree of anisotropy (DA) were inconsistent between the groups, and did not show significant correlations (*p* ≥ 0.109) between the methods (Figure 2j–l), thus, detection of these parameters with clinical CT was categorized as “unreliable” hereafter. The intertechnique differences of all parameters excluding the “unreliable” ones were smaller than their intergroup (mild‐to‐moderate vs severe OA) differences with clinical CT (Table S1, Supporting Information). Based on Bland–Altman plots, clinically measurable difference could be observed in a parameter where the methodical bias (cCT–µCT) was less than 50% of the difference between the two OA stages (sOA–mOA). According to these criteria, noncalcified cartilage thickness, calcified tissue thickness, BV/TV, BS/BV, SMI, and Tb.Sp showed clinically measurable difference in subchondral bone structural changes with clinical CT (Figure S2, Tables S1 and S2, Supporting Information).

Principal components analysis (PCA) and cluster analysis of the micro‐CT (Figure 2m) and clinical CT (Figure 2n) datasets were able to separate the two OA groups. One‐way analysis of similarities (ANOSIM) and one‐way permutational multivariate analysis of variance (PERMANOVA) exposed significant differences between the mild‐to‐moderate and severe OA groups with micro‐CT (*R* = 0.7207, *p* = 0.0001; and *F* = 21.19, *p* = 0.0002, respectively) and clinical CT too (*R* = 0.4449, *p* = 0.0003; and *F* = 14.91, *p* = 0.0005, respectively). Otsu's automatic thresholding provided highly similar results (Figure S3, Supporting Information) and examination of a tungsten wire phantom showed that clinical CT was able to visualize thin structures in the range of human trabecular thickness (Figure S4, Supporting Information). These data highlight the usability of both clinical and micro‐CT to accurately distinguish between mild‐to‐moderate and severe OA tibial plateaus based on trabecular microstructural changes. Moreover, the relative differences between clinical CT and micro‐CT in the present investigation were smaller than those reported in most of the other studies comparing clinical CT or MRI to the gold‐standard micro‐CT (Table S3, Supporting Information). The strong correlations and relatively small differences between the data acquired with the two CT methods support the usability of the clinical CT to monitor microstructural OA changes.

### 2.3 Most Clinical CT Parameters Show High Intra‐ and Interobserver Reliability

Intraobserver repeatability of almost all measurements was good or excellent,^[^ ^22^ ^]^ using micro‐CT (intraclass correlation coefficients, ICC ≥ 0.8227, in *n* = 12 of 13 tested parameters) (**Table** **1** and Figure S5, Supporting Information), and clinical CT (ICC ≥ 0.7977, in *n* = 11 of 13 tested parameters) (Table 1).

**Table 1:** Intra‐ and interobserver reliability of the measurements. Noncalcified cartilage and calcified tissue thicknesses were manually measured, and VOIs of the subarticular spongiosa were manually selected twice with 3.5 months difference by the same observer, or by three individual observers, and were evaluated automatically. ICCs were calculated with “absolute agreement,” and the results are given together with their 95% confidence intervals (CIs). Color codes indicate ICC reliability according to Koo et al.^[^ ^22^ ^]^ (red: poor <0.5; orange: 0.5 < moderate < 0.75; blue: 0.75 < good < 0.9; green: 0.9 <excellent)

	Micro‐CT		Clinical CT	
	ICC	95% CI	ICC	95% CI
Intraobserver comparison (1 observer, 2 trials)				
Noncalcified cartilage thickness	0.9895	0.9734–0.9958	0.9847	0.9625–0.9939
Calcified tissue thickness	0.9001	0.7590–0.9597	0.5242	−0.0590–0.8146
BV/TV	0.9167	0.5201–0.9754	0.9691	0.9177–0.9880
BS/BV	0.9394	0.8250–0.9772	0.9304	0.8355–0.9717
BS/TV	0.9251	0.6736–0.9757	0.9019	0.3427–0.9728
Tb.Pf	0.8692	0.4288–0.9585	0.9285	0.8310–0.9709
SMI	0.8429	0.3791–0.9487	0.8820	0.7306–0.9512
Tb.Th	0.9374	0.8086–0.9770	0.8657	0.6767–0.9458
Tb.N	0.9199	0.6467–0.9742	0.8988	0.4501–0.9698
Tb.Sp	0.8695	0.4906–0.9567	0.8596	0.6188–0.9462
FD	0.5565	0.1574–0.7981	0.3880	−0.0171–0.6953
Conn.Dn	0.9417	0.7752–0.9802	0.7977	0.0056–0.9447
DA	0.8227	0.6007–0.9263	0.9204	0.8096–0.9678
Interobserver comparison (3 observers)				
Noncalcified cartilage thickness	0.7711	0.5872–0.8929	0.8683	0.7307–0.9426
Calcified tissue thickness	0.7447	0.5510–0.8787	0.5669	0.1691–0.8074
BV/TV	0.9104	0.8223–0.9605	0.9725	0.9412–0.9883
BS/BV	0.9305	0.8616–0.9695	0.8859	0.7543–0.9515
BS/TV	0.7796	0.5761–0.9005	0.9340	0.8680–0.9711
Tb.Pf	0.9425	0.8841–0.9749	0.8866	0.7487–0.9525
SMI	0.9208	0.8424–0.9651	0.8607	0.7142–0.9394
Tb.Th	0.9106	0.8240–0.9604	0.8451	0.6164–0.9385
Tb.N	0.8138	0.6455–0.9156	0.9039	0.8109–0.9574
Tb.Sp	0.7254	0.4819–0.8745	0.8700	0.7518–0.9414
FD	0.6247	0.3493–0.8187	0.0499	−0.0520–0.2376
Conn.Dn	0.7802	0.6007–0.8977	0.8566	0.7291–0.9350
DA	0.9001	0.8055–0.9555	0.8638	0.7018–0.9426

Likewise, the measurements displayed mostly good or excellent interobserver repeatability too using micro‐CT (ICC ≥ 0.7711, in *n* = 10 of 13 tested parameters) (Table 1 and Figure S6, Supporting Information), and clinical CT (ICC ≥ 0.8451, in *n* = 11 of 13 tested parameters) (Table 1). Although Tb.Sp displayed only moderate interobserver reliability with micro‐CT (ICC = 0.7254), its other ICCs were good (≥ 0.8596). Only calcified tissue thickness and FD showed moderate or poor intraclass correlations (ICC ≤ 0.5669) multiple times using micro‐CT and clinical CT (Table 1). These two parameters were also classified as “unreliable” hereafter. These data underscore the excellent repeatability of nearly all manual image‐based measurements, both on clinical CT and micro‐CT image sets.

### 2.4 In Vivo Clinical CT Reliably Differentiates Normal and Mild‐to‐Moderate OA from Severe OA

**Figure 3:**
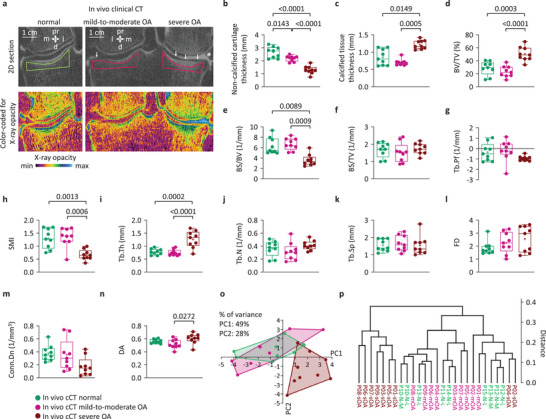
In vivo clinical CT detection of osteochondral parameters in normal, mild‐to‐moderate, and severe OA tibial plateaus. a) Clinical (arthro‐)CT scans of two representative patient knees, showing a normal medial, a mild‐to‐moderate OA lateral, and a severe OA medial compartment (arrows: cartilage thinning, asterisk: meniscal extrusion, polygons: analysis ROIs in the subarticular spongiosa). Box plots of the b) noncalcified cartilage thickness, c) calcified tissue thickness, subarticular spongiosa d) BV/TV, e) BS/BV, f) BS/TV, g) Tb.Pf, h) SMI, i) Tb.Th, j) Tb.N, k) Tb.Sp, l) FD, m) Conn.Dn, and n) DA. *n* = 9 per group; ANOVA or Kruskal–Wallis ANOVA. All subarticular spongiosa parameters were used as input data visualizing the separation of the samples into groups by o) PCA and p) cluster analysis. Data points represent individual normal, mild‐to‐moderate, and severe OA tibial plateaus (*n* = 9 per group). Information content (% of variance) of axes for principal components 1 (PC1) and 2 (PC2) are shown above graphs. Abbreviations: cCT, clinical CT.

Quantitative in vivo clinical (arthro‐)CT examination of normal and mild‐to‐moderate OA tibial plateaus (**Figure** **3**a and Table S4, Supporting Information) revealed significant difference in noncalcified cartilage thickness (*p* = 0.0143) (Figure 3b), but not in any bone structural parameters (*p* ≥ 0.3349) (Figure 3c–n). Most importantly, in vivo clinical CT comparison of normal and mild‐to‐moderate OA with severe OA (Figure 3a–n) revealed similar osteochondral differences as ex vivo imaging. These differences included significantly lower noncalcified cartilage thickness, BS/BV, and SMI, and higher calcified tissue thickness, BV/TV, Tb.Th, and DA in severe OA (all *p* ≤ 0.0272) (Figure 3b–n). A similar trend of differences as ex vivo was detected in Tb.Pf, Tb.N, Tb.Sp, and Conn.Dn (*p* ≥ 0.1206) (Figure 3b–n, and see also Figure S7, Supporting Information). Furthermore, PCA and cluster analysis, based on all subarticular spongiosa parameters, were able to separate severe from normal and mild‐to‐moderate OA (Figure 3o,p). ANOSIM and PERMANOVA did not reveal significant differences between normal and mild‐to‐moderate OA (both *p* ≥ 0.775), but exposed significant differences between normal and severe OA (*R* = 0.4201, *p* = 0.0003; and *F* = 8.378, *p* = 0.0012, respectively), and mild‐to‐moderate and severe OA (*R* = 0.6084, *p* = 0.0003; and *F* = 9.707, *p* = 0.0003, respectively). These findings indicate that, although mild‐to‐moderate OA could not be distinguished from normal, in severe OA patients characteristic quantitative microstructural osteochondral changes can be visualized by clinical (arthro‐)CT examinations.

### 2.5 Medium‐Resolution Micro‐CT Datasets are More Similar to Clinical CT than to High‐Resolution Micro‐CT Datasets

**Figure 4:**
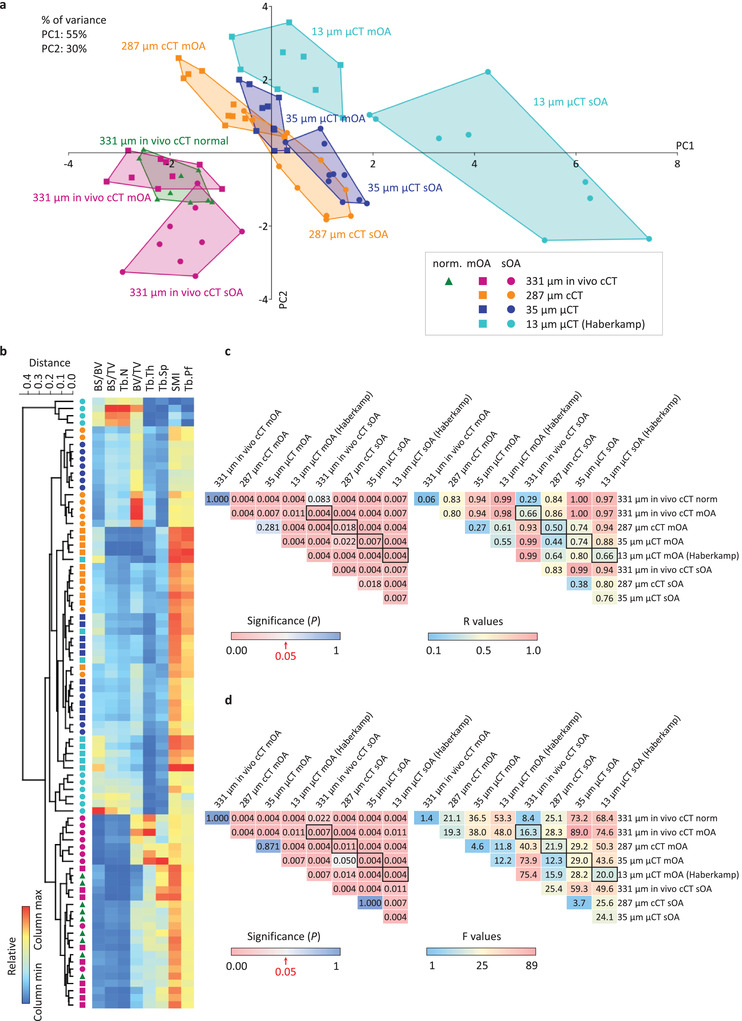
Multivariate comparison of 331, 287, 35, and 13 µm resolution CT techniques excluding the “unreliable” parameters. Raw data (BV/TV, BS/BV, BS/TV, Tb.Th, Tb.Sp, Tb.N, Tb.Pf, and SMI) from a previous study (Haberkamp et al. 2020)^[^ ^2e^ ^]^ were re‐evaluated together with the present study's data with a) PCA, b) cluster analysis, c) one‐way analysis of similarities (ANOSIM), and d) one‐way permutational multivariate analysis of variance (PERMANOVA), to compare the separation of the datasets corresponding to mild‐to‐moderate and severe OA groups, with in vivo clinical CT (331 µm resolution), ex vivo clinical CT (287 µm resolution), and micro‐CT (35 and 13 µm resolution). “Unreliable” parameters (calcified tissue thickness, FD, Conn.Dn, and DA) were excluded from the analysis. Higher *R* and *F* values indicate larger difference between the groups. Framed cells designate comparisons between mild‐to‐moderate and severe OA with identical CT techniques. Abbreviations: mOA, mild‐to‐moderate OA; sOA, severe OA; cCT, clinical CT; *μ*CT, micro‐CT.

Next, different resolution (13,^[^ ^2e^ ^]^ 35, 287, and 331 µm) CT datasets were compared to assess their performance to differentiate between mild‐to‐moderate and severe OA and to examine the intertechnique differences. In the multivariate analyses, the “unreliable” parameters (calcified tissue thickness, FD, Conn.Dn, and DA) were excluded, and the present study's data were compared with published datasets.^[^ ^2e^ ^]^ In vivo clinical CT with 331 µm, ex vivo clinical CT with 287 µm, and micro‐CT with 35 and 13 µm^[^ ^2e^ ^]^ resolutions were all able to differentiate mild‐to‐moderate OA from severe OA with PCA (**Figure** **4**a), cluster analysis (Figure 4b), ANOSIM and PERMANOVA (all *p* ≤ 0.018, *R* ≥ 0.50, *F* ≥ 16.3, respectively) (Figure 4c,d).

Ex vivo 287 µm clinical CT showed higher similarity with 35 µm (medium resolution) micro‐CT (ANOSIM *p* = 0.281 and 0.018, *R* ≤ 0.38; and PERMANOVA *p* ≥ 0.871, *F* ≤ 4.6) (Figure 4c,d) than 35 µm micro‐CT with 13 µm (high resolution) micro‐CT (ANOSIM *p* ≤ 0.007, *R* ≥ 0.55; and PERMANOVA *p* ≤ 0.007, *F* ≥ 12.2) (Figure 4c,d) both in mild‐to‐moderate and severe OA (see also Figures S8 and S9, Supporting Information). Furthermore, the intertechnique differences between 287 µm clinical CT and 35 µm micro‐CT were smaller than the intergroup differences between mild‐to‐moderate and severe OA (Figure 4c,d). These results highlight the capability of all in vivo and ex vivo CT techniques to differentiate normal or mild‐to‐moderate OA from severe OA, and the high similarity of the ex vivo 287 and 35 µm scans, validating clinical CT for a quantitative monitoring of microstructural OA changes both in clinical and laboratory settings. These data also validate the finding that solely using the 3D subchondral bone microstructural parameters can differentiate between the two stages of OA without considering any other structural indicators.

### 2.6 Clinical CT Reliably Detects Fundamental Subchondral Bone Correlations

**Figure 5:**
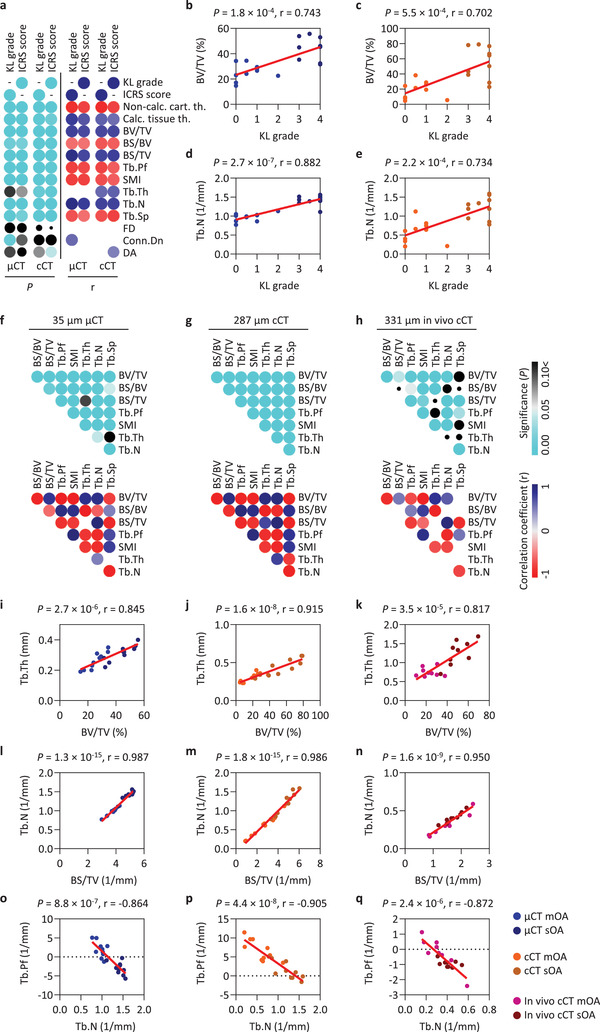
Correlation to clinical gold standard, and correlation of the regional patterns of the “reliable” subchondral bone parameters. a) Pearson correlation matrices of the ex vivo micro‐CT and clinical CT parameters with the mean KL grades and ICRS scores. Correlation matrix of the *p*‐values; cyan dots show significant (*p* < 0.05) correlations, the decreasing size of dots indicates the increase of the *p* values. Correlation coefficients (*r*) of the significant (*p* < 0.05) correlations. Scatter plot and linear regression of correlation between b,c) KL grades and BV/TV, and d,e) KL grades and Tb.N. Pearson correlation matrices of the “reliable” subchondral bone microstructural parameters detected with f) micro‐CT with 35 µm, g) ex vivo clinical CT with 287 µm, and h) in vivo clinical CT with 331 µm resolutions. Scatter plot and linear regression of correlation between i–k) BV/TV and Tb.Th, l–n) BS/TV and Tb.N, and o–q) Tb.N and Tb.Pf derived from the different resolution CT techniques. Abbreviations: mOA, mild‐to‐moderate OA; sOA, severe OA; cCT, clinical CT; *μ*CT, micro‐CT.

To examine the association of subchondral bone microstructure to the stage of OA, Pearson correlations with KL grades and ICRS scores were examined (**Figure** **5**a–e and Table S5, Supporting Information). Significant moderate to very strong (*r* ≤ −0.521 or ≥ 0.562, *p* ≤ 0.018) associations were observed in most of the “reliable” parameters (except Tb.Th) with both CT techniques. For example, the strong correlations (*r* ≥ 0.702, *p* ≤ 5.5 × 10^−4^) between the KL grades and BV/TV (Figure 5b,c) or Tb.N (Figure 5d,e) expose the expansion of the subchondral trabecular structure with the aggravation of OA.

Finally, as a case study, the ability of clinical CT was examined to detect the fundamental subchondral bone correlations described earlier in varus OA.^[^ ^2e^ ^]^ Pearson correlation matrices of the regional patterns of the “reliable” parameters were compared between micro‐CT with 35 µm, ex vivo clinical CT with 287 µm, and in vivo clinical CT with 331 µm resolutions (Figure 5f–h). Out of *n* = 28 tested comparisons *n* = 26 (Figure 5f), *n* = 28 (Figure 5g), and *n* = 19 (Figure 5h), respectively, showed significant correlations (*p* < 0.05), indicating that all techniques are able to detect important strong associations between the parameters.

The very strong correlation between BV/TV and Tb.Th (*r* ≥ 0.817, *p* ≤ 3.5 × 10^−5^) (Figure 5i–k) mirrored the increased relative bone volume in an increased trabecular thickness, and the one between Tb.N and BS/TV (*r* ≥ 0.950, *p* ≤ 1.6 × 10^−9^) (Figure 5l–n) represented the parallel increased number of the trabeculae and relative bone surface in the locations more severely affected by OA. Furthermore, the very strong, negative correlation between Tb.N and Tb.Pf (*r* ≤ −0.864, *p* ≤ 2.4 × 10^−6^) (Figure 5o–q) denoted an increased number and enhanced connectivity of the trabeculae in more severe OA. These analyses suggest that the complex associations between the microstructural changes caused by subchondral bone remodeling during the progression of human knee OA can be detected by all evaluated CT techniques, emphasizing the usability of clinical CT for OA research and therapy.

## 3 Discussion

The findings of this study provide evidence that clinical CT could be used to monitor microstructural changes as an advanced tool to help human OA diagnosis, treatment, and clinical investigations. First, clinical CT reliably discriminates between mild‐to‐moderate and severe cases of OA in vivo when using quantitative 3D structural osteochondral parameters. Second, detection of most parameters (noncalcified cartilage thickness, BV/TV, BS/BV, BS/TV, Tb.Pf, SMI, Tb.Th, Tb.N, and Tb.Sp) shows significant and strong correlations with micro‐CT, a high intra‐ and interobserver reliability, and moderate to very strong correlations with clinical OA grades, and are thus usable for clinical and experimental assessments of microstructural subchondral bone changes. Third, the striking similarity of the results obtained with clinical CT at 287 µm and micro‐CT at 35 µm resolutions challenges the view that structural measurements using clinical CT with a resolution near the trabecular dimensions are inaccurate. Fourth, considering solely the 3D subchondral bone microstructural parameters allows to differentiate between mild‐to‐moderate and severe OA (without examining other parameters).

Subchondral bone plate sclerosis and degradation of subarticular trabeculae are hallmarks of microstructural bone changes in OA, together with the development of osteophytes, bone marrow lesions, and subchondral cysts.^[^ ^2^ ^]^ In this bone remodeling process, osteocytes play a crucial role by activating osteoclasts via releasing chemokines, adenosine triphosphate, membrane‐derived lipids, and receptor activator of NF*κ*B ligand.^[^ ^23^ ^]^ The development of osteophytes and cysts is facilitated by growth factors, among which transforming factor‐*β* (TGF*β*).^[^ ^23^, ^24^ ^]^ These structural alterations modify local shear stresses, causing cartilage to distort and split, while pathological molecular and biochemical cross‐talk among the cells of the osteochondral unit also participate in aggravation of the disease.^[^ ^23^ ^]^ Both clinical CT and micro‐CT detected significantly increased calcified tissue thickness, BV/TV, BS/TV, Tb.Th, and Tb.N, and decreased noncalcified cartilage thickness, BS/BV, Tb.Pf, SMI, and Tb.Sp in severe OA. These parameters showed strong significant correlations between the two measurement systems (except FD, Conn.Dn, and DA). In vivo clinical CT differentiated between alterations of hip trabecular microstructure with high intra‐ and interobserver reliability.^[^ ^25^ ^]^ Strong correlations between ex vivo subchondral bone parameters of other joints assessed have been reported at lesser detail.^[^ ^26^ ^]^ Here, measurements with both CT methods showed high intra‐ and interobserver reliability (except for calcified tissue thickness and FD). Important associations^[^ ^2e^ ^]^ between the “reliable” subchondral bone microstructural parameters and with clinical OA grades were detected with both methods, suggesting that the complex spatiotemporal bone remodeling processes which accompany knee OA progression are traceable with CT imaging both in laboratory and clinical conditions. Furthermore, these trabecular microstructural differences are suitable for distinguishing between mild‐to‐moderate and severe OA without considering any other classical OA markers among which osteophytes, joint space narrowing, subchondral bone cysts, or cartilage damage.

The novelty of the present study is that both clinical and micro‐CT datasets were evaluated identically, applying the same precise 3D micro‐CT analysis method. Previously, clinical CT data were analyzed with algorithms different from for the micro‐CT data, possibly reducing comparability. Due to the precise 3D evaluations, relative differences between the values of most parameters measured with the two CT techniques were considerably smaller than those reported when comparing micro‐CT versus MRI,^[^ ^27^ ^]^ comparable or smaller than in other micro‐CT versus clinical CT comparisons,^[^ ^26^, ^27^, ^28^ ^]^ and comparable to the considerably higher resolution (isotropic 82 µm) HR‐pQCT^[^ ^26e^ ^]^ (Table S3, Supporting Information), also supporting the notion that clinical CT resolves better mineralized tissue structure than MRI.^[^ ^10^ ^]^ Resolution‐dependent differences can be explained by the partial volume effect, reduced surface detail, artifactual merging of adjacent trabeculae or loss of thin trabeculae at the higher voxel size,^[^ ^29^ ^]^ and anisotropic voxels of the clinical CT. However, the clinical CT datasets were less sensitive to such effects than artificially reduced resolution micro‐CT image sets (Figure S10, Supporting Information). The limited ability of clinical CT to detect the thinnest trabecular elements may cause a minimal overestimation of the mean Tb.Th, and a minimal underestimation of BV/TV, BS/TV, BS/BV, and Tb.N. This reduced information still allows clinically meaningful differentiation between mild‐to‐moderate and severe OA by clinical CT, solely based on the structural alterations of the larger trabeculae, encouraging its usability in clinical and experimental settings. Thus, accuracy of clinical CT is excellent to quantitatively determinate subchondral bone microstructure of the knee compared to all other available techniques. However, clinical CT should not primarily be considered as an advanced imaging tool in daily practice (e.g., considering radiation exposure or expenditure) capable of replacing X‐ray or MRI, but rather to better understand mechanisms of human knee OA in special settings.^[^ ^10^ ^]^ For example, it may be applied in randomized clinical trials that investigate novel compounds targeting the subchondral bone in OA, and, bearing in mind its limitations, also in preclinical or ex vivo studies, e.g., relating such microstructural data to histopathological findings.

The patterns of subchondral bone microstructural changes characteristic of distinct OA phenotypes were comparable between clinical and micro‐CT, and clearly usable for separating mild‐to‐moderate and severe OA phenotypes with all examined resolutions. The higher similarity of the results between clinical CT and medium‐resolution micro‐CT scans, than between the medium‐ and high‐resolution micro‐CT scans suggests that structural measurements with a resolution near the trabecular dimensions can be accurate despite earlier concerns.^[^ ^10^ ^]^ The large difference between 35 and 13 µm resolution micro‐CT measurements accentuates the need for standardizing the validation protocols, considering that various resolutions between 8 and 50 µm,^[^ ^26^, ^27^ ^]^ even up to 100 µm,^[^ ^28^ ^]^ are frequently used as gold‐standard for validating clinical CT and MRI (Table S3, Supporting Information). In vivo clinical CT revealed similar quantitative osteochondral differences between normal or mild‐to‐moderate OA versus severe OA as ex vivo measurements. Multivariate analyses validated its capability to differentiate between normal or mild‐to‐moderate OA versus severe OA groups. These findings also show an unexpected accuracy and usability of the measurements even in the resolution range of trabecular dimensions, opening a door to quantitative applications of clinical CT.

Limitations include that partial volume effect and anisotropic voxels may render the computing of certain fine trabecular microstructural parameters (FD, Conn.Dn, and DA) unreliable with clinical CT, yet the majority of structural measures strongly correlated with micro‐CT and the global phenotypic differences between mild‐to‐moderate and severe OA were safely distinguishable. The challenging manual measurement of human calcified tissue thickness may be improved using automatic VOI selection and fitting algorithms.^[^ ^30^ ^]^ Based on previous data,^[^ ^26^, ^28^ ^]^ FD, Conn.Dn, and DA cannot be reliably assessed with clinical CT and need to be measured in vivo with HR‐pQCT.^[^ ^26e^ ^]^ Nevertheless, most studies^[^ ^26^, ^27^ ^]^ report chiefly BV/TV, Tb.Th, Tb.Sp, and Tb.N, and our data show these can reliably characterize mild‐to‐moderate and severe OA when supplemented with BS/TV, BS/BV, Tb.Pf, and SMI. Strengths include a highly standardized experimental protocol, the same set of human samples for micro‐CT and clinical CT acquisitions at identical anatomic locations, the matching image processing procedures, allowing for a direct comparison, together with the inter‐ and intraobserver comparison, and validation with clinical datasets.

In the future, newer generation scanners or specialized devices similar to HR‐pQCT^[^ ^26e^ ^]^ will allow even higher resolution at lower radiation doses. Applying such structural bone measures will further improve our understanding of OA, and may contribute to the development of reliable machine learning‐based methods^[^ ^31^ ^]^ by implementing precise automatic VOI selection and thresholding algorithms that require minimal operator input.^[^ ^30^ ^]^ Detection of microstructural OA alterations by clinical CT could also implement deep learning and artificial intelligence tools, including fully automated knee tissue segmentation, disease characterization with KL grades, and identification of structural joint pathologies with excellent diagnostic performance, as with other techniques.^[^ ^6^ ^]^ Clinical studies are now warranted to define the range of subchondral bone parameters in different stages of OA or other structural bone diseases by clinical CT, among which osteoporosis, e.g., in multi‐center trials and big data analyses and finally testing its broad application and exact sensitivity, specificity and accuracy in a cross‐sectional study population. Extended CT quantification of hip OA might be also envisaged^[^ ^25^ ^]^ respecting joint‐specific limitations such as a possibly lower accuracy due to thinner trabeculae, X‐ray attenuation by soft tissues, and the risk of X‐ray exposure of nearby organs. Some variation in the detected parameters across different clinical CT devices may be expected, as also reported for different micro‐CT manufacturers and analysis programs (Table S6, Supporting Information).

In sum, 3D subchondral bone microstructure reveals moderate to very strong correlations with mild‐to‐moderate and severe cases of OA. Clinical CT measurements show strong correlations with the gold standard micro‐CT, high inter‐ and intraobserver reliability, and comparable or even minor relative differences than other imaging methods.^[^ ^26^, ^27^ ^]^ Clinical CT therefore offers an opportunity to quantitatively monitor subchondral bone microstructure in clinical and experimental settings as an advanced tool of investigating OA and other diseases affecting bone architecture.

## 4 Experimental Section

### Study Design

Human proximal tibia samples (*n =* 10), derived from total knee replacements, displaying medial or lateral compartment dominant advanced OA, were scanned with clinical CT (287 µm resolution) and micro‐CT (35 µm resolution). Although OA affected distal femora similarly, only tibial plateaus were studied due to their anatomical landmarks allowing a more reproducible VOI positioning,^[^ ^18^ ^]^ and availability of sufficiently thick samples for reliable subchondral analyses. Due to the specific nature of the required samples, randomization was not possible. Sample size requirements were determined based on previous studies.^[^ ^2d,e^ ^]^ The medial and lateral tibial plateaus (total *n* = 20) were scored separately by the ICRS macroscopic scoring system, and classified into two groups: mild‐to‐moderate OA (*n* = 10), and severe OA (*n* = 10). Knees of OA patients (*n* = 9) were scanned with in vivo clinical (arthro‐)CT (331 µm resolution), and the medial and lateral tibial plateaus were divided into mild‐to‐moderate (*n* = 9) and severe OA (*n* = 9) groups. Clinical (arthro‐)CT scans of tibial plateaus (*n* = 9) of patients without radiographic or symptomatic knee OA were used as normal controls. The resolution of clinical CT scans was selected to maximize the translational comparability to micro‐CT ex vivo (287 µm), and to faithfully reflect the everyday clinical situation in vivo (331 µm). In each clinical CT and micro‐CT image sets, noncalcified cartilage thickness and calcified tissue (including subchondral bone plate and calcified cartilage) thickness were measured manually, and VOIs were selected manually in the subarticular spongiosa. Manual measurements and VOI selection in the ex vivo datasets were repeated by two other independent observers, and 3.5 months later by the first observer to test inter‐ and intraobserver reliability. To obtain accurate 3D subchondral bone microstructural parameters and directly comparable results, VOIs scanned by both CT techniques were evaluated with the same micro‐CT analysis software. The well‐documented structural differences between mild and severe OA^[^ ^2^ ^]^ were sought after to validate clinical CT with micro‐CT. To examine the dependency of the parameters on resolution, the data were compared with a 13 µm resolution micro‐CT dataset of a previous study^[^ ^2e^ ^]^ by multivariate analyses.

### Ex Vivo Human Samples and Radiographs

Human late OA proximal tibia samples (*n* = 10; including 4 females and 6 males; 5 left and 5 right knees, 4 with varus and 6 with valgus malalignment; mean age: 64 ± 12 years) were obtained as surgical discards from the Department of Orthopedic Surgery, Saarland University Medical Center from patients undergoing total knee replacement. Only samples containing both the medial and lateral tibial plateaus, without visible cutting artifacts were used for analysis, with an adequate thickness (≥5 mm) of the subarticular spongiosa allowing reliable bone microstructural results. Preoperative anteroposterior radiographs of each knee joint (*n* = 10) were acquired using a Siemens Arcadis Varic image intensifier (Siemens) and were graded according to the semiquantitative KL grading system^[^ ^20^ ^]^ by two blinded observers (HM and TO). Informed consent was obtained from all participants. The study was approved by the Ethics Committee of the Saarland Physicians Council (*Ärztekammer des Saarlandes, Ethik‐Kommission*, No. 267/17).

### Macroscopic Scoring and Classification of the Tibial Plateaus

Each ex vivo proximal tibia were divided into medial (*n* = 10) and lateral (*n* = 10) tibial plateaus, and an ≈1 cm long (antero‐posterior) region of interest (ROI) between the tibial spines and the outer edge of the plateau was evaluated (Figure 1a). These ROIs were scored by two blinded observers (HM and TO) based on the extent of OA using the International Cartilage Regeneration and Joint Preservation Society (ICRS) grading system,^[^ ^19^ ^]^ adapted to the tibial plateaus as reported previously,^[^ ^2d^ ^]^ not providing information about the distal femora. ROIs with severe cartilage degradation, penetrating into the subchondral bone (i.e., denudation; ICRS score 4), were classified as severe OA (*n* = 10 including *n* = 6 lateral and *n* = 4 medial tibial plateaus of distinct patients, ICRS score 4 ± 0), while ROIs with cartilage damage not reaching the subchondral bone (ICRS scores ≤ 3) were classified as mild‐to‐moderate OA (*n* = 10 including *n* = 4 lateral and *n* = 6 medial tibial plateaus of distinct patients, ICRS score 2.25 ± 0.64; Figure 1a). Macroscopic extent of denudation within the ROIs was measured with ImageJ (v. 1.51, National Institutes of Health, MD, USA). As a result, in each proximal tibia, one tibial plateau (either the medial or the lateral ROI) was classified as “severe,” and the other one as “mild‐to‐moderate” OA, allowing for paired comparisons between the groups (technical repeats were one scan per group per patient per technique).

### Ex Vivo Clinical CT Imaging

The *n* = 10 whole human proximal tibia specimens were scanned in a clinical multidetector CT scanner with 128 rows (SOMATOM Definition Edge 128, Siemens), routinely used for examining patients at Clinique d'Eich, Centre Hospitalier de Luxembourg, Eich, Luxembourg (tube voltage, 120 kV; current, 169 mA; 16 rows acquisition). Coronal images were created (Kernel Ur 68 for bone) with a matrix size of 512 × 512 pixels and a field of view of 132 × 50 mm resulting in a final nominal in‐plane spatial resolution of 0.287 mm. Slice thickness of the reconstructed coronal images was set to 1 mm. Image stacks were then exported as “dicom” archives with RadiAnt DICOM Viewer v.2020.2. (Medixant, Poznan, Poland) and evaluated with CTAnalyzer (v. 1.16.4.1, Bruker micro‐CT, Kontich, Belgium).

### In Vivo Clinical CT (arthro‐CT) Imaging

Informed consent was obtained from the patients to the use of the arthro‐CT images. The study was conducted according to the rules of the Declaration of Helsinki and in compliance with all relevant ethical regulations of the Saarland University and the Clinique d“Eich regarding the use of human study participants. Patients with varus OA knees (*n* = 9; including 1 female and 8 males; 4 left and 5 right knees; mean age: 61 ± 10 years; divided into *n* = 9 severe OA medial and *n* = 9 mild‐to‐moderate OA lateral tibial plateaus), and normal patients with meniscal pain, ligament distortion, or patella impingement, but without any radiographic signs or reported symptoms of OA (*n* = 9, from 2 left and 4 right knees of 6 male patients, including 6 lateral and 3 medial tibial plateaus; mean age: 57 ± 11 years) (technical repeats were one scan per patient) at the Clinique d”Eich, Luxembourg were examined with the same clinical multidetector CT scanner as used for the ex vivo experiments (SOMATOM Definition Edge 128, Siemens; tube voltage, 120 kV; current, 152 mA; 16 rows acquisition) after receiving a contrast medium (Omnipaque 300; GE Healthcare) via intra‐articular injection. The effective dose of radiation was 0.1 mSv, similar to that of a retour transatlantic flight.^[^ ^32^ ^]^ Coronal images were created (Kernel Ur 68 for bone) with a matrix size of 512 × 512 pixels and a field of view of 20 cm resulting in a mean final nominal in‐plane spatial resolution of 0.331 mm and a slice thickness of 1 mm. Image stacks were then exported as “dicom” archives with RadiAnt DICOM Viewer and evaluated with CTAnalyzer identically to the ex vivo scans.

### Ex Vivo Micro‐CT Imaging

The same set of *n* = 10 whole human proximal tibia specimens was scanned in a micro‐CT scanner (SkyScan 1176, Bruker micro‐CT; nominal isotropic voxel size, 35 µm; tube voltage, 75 kV; current, 291 µA; combined 0.5 mm aluminum/copper filter; 0.4° intervals; 270 ms exposure time, 3 averaging frames). Reconstruction was performed by a modified Feldkamp cone‐beam algorithm^[^ ^33^ ^]^ with NRecon software (v. 1.7.0.4, Bruker micro‐CT). To obtain comparable image sets, the reconstructed micro‐CT datasets were rotated uniformly (DataViewer software v. 1.5.2.4., Bruker microCT) prior to saving the coronal section images for further evaluations.^[^ ^2^, ^18^ ^]^ Reproducibility of micro‐CT measurements was confirmed previously by repeated measurements of human tibial plateau samples, resulting in similar values of all tested parameters.^[^ ^2d^ ^]^

### Evaluation of the CT Data

Ex vivo clinical CT and micro‐CT (Figure S1, Supporting Information), and in vivo clinical CT datasets were evaluated similarly. In the subarticular spongiosa of the mild‐to‐moderate and severe OA ROIs ≈3 mm deep (downward from the visually determined bottom of the subchondral bone plate at the beginning of the marrow space^[^ ^34^ ^]^) and 1 cm long (antero‐posterior) VOIs were selected manually. Evaluation threshold for subchondral bone was predetermined for each subarticular spongiosa VOI by histogram analysis on a specimen‐by‐specimen basis with Otsu's automatic thresholding^[^ ^35^ ^]^ (resulting in different threshold values for every VOI of each sample), then the mean threshold values of all in vivo (85 and 255; *n* = 27) and ex vivo (92 and 255; *n* = 20) clinical CT, and micro‐CT (72 and 255; *n* = 20) datasets were used as global thresholding values. The following parameters were determined in all clinical CT and micro‐CT subarticular spongiosa VOIs using the software CTAnalyzer (v. 1.16.4.1, Bruker micro‐CT): percent bone volume (BV/TV; relative volume of calcified tissue in the selected volume of interest), bone surface‐to‐volume ratio (BS/BV; a measure for the bone surface per given bone volume), bone surface density (BS/TV; ratio of surface area to total volume), trabecular thickness (Tb.Th; thickness of the trabecular structure), trabecular separation (Tb.Sp; thickness of the spaces between the trabeculae), trabecular number (Tb.N; inverse of the mean distance between the mid‐axes of the examined structure), trabecular pattern factor (Tb.Pf; a parameter of cancellous bone connectivity), structure model index (SMI; shows the relative prevalence of plates and rods), degree of anisotropy (DA; a measure of how highly oriented substructures are within a volume), fractal dimension (FD; an indicator of surface complexity), and connectivity density (Conn.Dn; characterizes the redundancy of trabecular connections).^[^ ^2d^ ^]^ For simplicity and because they were computed identically (same analyzing program and algorithms) to those of the corresponding micro‐CT image sets, we chose not to label the morphological parameters deriving from the lower resolution^[^ ^36^ ^]^ and nonisotropic clinical CT image sets distinctly as “apparent.” For 3D reconstruction of the clinical CT and micro‐CT image sets, the CTVox v. 3.2.0 (Bruker micro‐CT) program was used, employing shadows and surface lighting to enhance the visibility of the surface structures of the samples.^[^ ^2d^ ^]^

Calcified tissue thickness was defined as including both the subchondral bone plate and the calcified cartilage that highly interdigitates with the underlying subchondral bone through its interface, the cement line; reflecting the inability of both CT techniques to precisely distinguish between these zones^[^ ^37^ ^]^ (Figure 1i). For manual measurements (unaffected by the thresholding) of the calcified tissue and noncalcified cartilage thickness, each VOI was divided into three thirds (outer, middle, inner), and thicknesses were measured in the most anterior, middle, and most posterior images of each VOI (*n* = 18 measurement points per tibial plateau), similarly in the clinical CT and micro‐CT datasets.

All ex vivo image‐based manual measurements and VOI selections were performed by three independent, blinded observers (TO, LG, and XC) to test interobserver reliability, and were repeated after 3.5 months by one observer (TO) to test intraobserver reliability. All other measurements were performed by a single observer (TO).

### Comparison with a Micro‐CT Dataset of a Previous Study

To compare the present results with higher resolution (13 µm) micro‐CT, raw data of human advanced varus knee OA medial (severe OA) and lateral (mild‐to‐moderate OA) tibial plateau samples (*n* = 9 each; *n* = 5 females, *n* = 4 males, mean age: 66.6 ± 8.7 years), scanned with micro‐CT (Skyscan 1172, Bruker microCT; isotropic resolution 13 µm, 70 kV, 139 µA, 0.5 mm Cu+Al filter, manual VOI selection, global thresholding) were re‐analyzed from the study of Haberkamp et al. in 2020.^[^ ^2e^ ^]^ Parameters missing from this dataset (noncalcified cartilage thickness and Conn.Dn), and the ones found to be “unreliable” by the analyses described later (calcified tissue thickness, DA, FD, and Conn.Dn), were excluded from the multivariate analyses.

### Statistical Analysis

Normal distribution and equal variance of the data were tested with the Shapiro–Wilk normality test and *f*‐test, respectively. When matching mild‐to‐moderate and severe OA (medial and lateral) tibial plateaus of the same knees, or matching clinical CT and micro‐CT measurements of the same samples were compared, depending on a normal distribution, statistical significance was tested by Student's paired *T*‐test (two‐tailed) or Wilcoxon signed‐rank test. When multiple groups or multiple subregions were compared, one way analysis of variance (ANOVA) followed by Tukey test or Kruskal–Wallis one way ANOVA followed by Dunn's test for all pairwise multiple comparisons was used, depending on the normal distribution of the data, and multiplicity adjusted *p* values were reported (default in Graphpad Prism; https://www.graphpad.com/guides/prism/latest/statistics/stat_multiplicity_adjusted_p_values.htm).

To test correlation between the examined variables, the Pearson correlation coefficient was calculated. To test the intra‐ and interobserver reliability of the image‐based manual measurements, ICCs were determined^[^ ^18^ ^]^ using the following settings: two‐way model, the same raters for all subjects, type: absolute agreement. All correlation coefficients were interpreted according to Evans^[^ ^21^ ^]^ (0.00–0.19 very weak; 0.20–0.39 weak; 0.40–0.59 moderate; 0.60–0.79 strong; 0.80–1.00 very strong) and all ICCs were interpreted according to Koo and Li^[^ ^22^ ^]^ (0.0–0.5 poor; 0.5–0.75 moderate, 0.75–0.9 good, 0.9–1.0 excellent).

PCA and hierarchical cluster analysis were performed for all calculated parameters of the subarticular spongiosa (BV/TV, BS/BV, BS/TV, Tb.Th, Tb.Sp, Tb.N, Tb.Pf, SMI, Conn.Dn, DA, and FD; excluding Conn.Dn, DA, and FD when comparison with datasets of a previous study was performed) as described earlier.^[^ ^2^, ^38^ ^]^ To quantitatively define intergroup similarities and differences, nonparametric ANOSIM and PERMANOVA with Gower's similarity index were computed, and considering multiplicity issues, the Bonferroni‐corrected *p* values were reported.^[^ ^2e^ ^]^ Heat maps were created with Morpheus (https://software.broadinstitute.org/morpheus).

All calculations were performed with Prism, version 8.2.1 (Graphpad Software, Inc., San Diego, USA), MedCalc, version 14.8.1 (MedCalc Software, Ostend, Belgium), and Past, version 4.04;^[^ ^38^ ^]^ a *p* < 0.05 was considered statistically significant. Data were expressed as mean ± standard deviation (SD). Box plot diagrams always showed the individual data points (dots), mean (+), minimum and maximum (whiskers), and the 75th and 25th percentiles (borders of the boxes). Raw data of the figures are included in Data S1 in the Supporting Information.

## Conflict of Interest

The authors declare no conflict of interest.

## Author Contributions

H.M. and T.O. designed the study; T.O. acquired micro‐CT data; F.W. acquired clinical CT data; T.O., L.G., and X.C. analyzed data; T.O. prepared the figures; T.O., D.P., M.C., and H.M. interpreted the data; T.O. and H.M. wrote the initial draft. All authors contributed to editing and revising the manuscript, and have approved the submitted version of the manuscript.

## Supporting information

Supporting Information

## Data Availability

All relevant data are included in the manuscript and its Supporting Information.
