# Supplementary material for: Quantifying the Human Subchondral Trabecular Bone Microstructure in Osteoarthritis with Clinical CT
Source: Adv Sci (Weinh). 2022 Jun 7;9(23):2201692. doi: 10.1002/advs.202201692 (PMC9376842; doi:10.1002/advs.202201692)
Supplement: Supplementary file 1 — Supporting Information [file ADVS-9-2201692-s001.pdf]

ADVANCED SCIENCE
Open Access

Supporting Information

for *Adv. Sci.*, DOI 10.1002/advs.202201692

Quantifying the Human Subchondral Trabecular Bone Microstructure in Osteoarthritis with Clinical CT

*Tamás Oláh, Xiaoyu Cai, Liang Gao, Frédéric Walter, Dietrich Pape, Magali Cucchiarini and Henning Madry**

Supporting Information

Quantifying the human subchondral trabecular bone microstructure in osteoarthritis with clinical CT

Tamás Oláh, Xiaoyu Cai, Liang Gao, Frédéric Walter, Dietrich Pape, Magali Cucchiarini, Henning Madry[*]

Table S1. Inter-technique and inter-group comparisons of the mean values of micro-CT (µCT) and clinical CT (cCT) measurements. **(I.)** Inter-technique (µCT vs. cCT) comparison of all evaluated samples (n = 20; mild-to-moderate and severe OA pooled together), with their 95% confidence intervals (CI). In the "Absolute difference" column, the mean absolute differences between µCT and cCT (calculated as cCT - µCT) are shown with their 95% CI. In the "Relative (factor)" column, the cCT data were expressed as a relative difference quotient of the µCT data (calculated as cCT/µCT) and shown with their 95% CI. Inter-group [mild-to-moderate (mOA) vs. severe OA (sOA); n = 10 samples each] comparisons with **(II.)** micro-CT and **(III.)** clinical CT, with their 95% CI. In the "Absolute difference" column, the mean absolute differences between sOA and mOA (calculated as sOA - mOA) are shown with their 95% CI. In the "Relative (factor)" column, the sOA data were expressed as a relative difference quotient of the mOA data (calculated as sOA/mOA) and shown with their 95% CI. *P* values were determined with paired T-test or Wilcoxon test. Significant ($P < 0.05$) differences are indicated with bold numbers.

**I. Inter-technique comparison using all samples**										
		**Micro-CT**		**Clinical CT**		***P***	**Differences**			
parameter	unit	mean	95% CI	mean	95% CI		Absolute difference	95% CI	Relative (factor)	95% CI
Non-calcified cartilage thickness	mm	1.67	1.14 - 2.21	1.51	1.04 - 1.99	**0.0136**	-0.16	-0.28 - -0.04	0.93×	0.82 - 1.04
Calcified tissue thickness	mm	0.84	0.69 - 0.99	0.94	0.82 - 1.05	0.055	0.10	0.00 - 0.20	1.18×	1.05 - 1.31
BV/TV	%	34.92	29.16 - 40.67	36.68	25.08 - 48.29	0.5644	1.77	-4.54 - 8.07	0.94×	0.76 - 1.12
BS/BV	1/mm	13.43	11.77 - 15.08	12.38	10.46 - 14.3	**0.0137**	-1.04	-1.84 - -0.24	0.91×	0.84 - 0.98
BS/TV	1/mm	4.32	3.98 - 4.65	3.64	2.92 - 4.37	**0.016**	-0.67	-1.21 - -0.14	0.82×	0.69 - 0.96
Tb.Pf	1/mm	-0.53	-1.99 - 0.93	4.06	2.31 - 5.81	**<0.0001**	4.59	3.96 - 5.22	-0.33×	-2.69 - 2.03
SMI		0.69	0.28 - 1.09	1.16	0.89 - 1.43	**<0.0001**	0.47	0.28 - 0.66	0.59×	-2.88 - 1.7
Tb.Th	mm	0.29	0.26 - 0.32	0.37	0.32 - 0.42	**<0.0001**	0.08	0.05 - 0.12	1.28×	1.19 - 1.38
Tb.N	1/mm	1.19	1.07 - 1.31	0.90	0.69 - 1.1	**0.0001**	-0.30	-0.43 - -0.17	0.72×	0.6 - 0.85

Tb.Sp	mm	0.59	0.54 - 0.64	0.58	0.48 - 0.68	0.8776	-0.01	-0.09 - 0.07	0.97×	0.84 - 1.11
FD		2.65	2.63 - 2.66	1.97	1.74 - 2.2	**<0.0001**	-0.68	-0.91 - -0.45	0.74×	0.66 - 0.83
Conn.Dn	1/mm^3	7.44	6.36 - 8.51	1.14	0.90 - 1.39	**<0.0001**	-6.30	-7.31 - -5.29	0.16×	0.12 - 0.2
DA		0.29	0.23 - 0.35	0.91	0.90 - 0.91	**<0.0001**	0.62	0.56 - 0.67	4.09×	2.73 - 5.45

II. Inter-group comparison with micro-CT

		**mOA**		**sOA**		***P***	**Differences**			
parameter	unit	mean	95% CI	mean	95% CI		Absolute difference	95% CI	Relative (factor)	95% CI
Non-calcified cartilage thickness	mm	2.69	2.35 - 3.04	0.66	0.3 - 1.02	**0.002**	-2.04	-2.38 - -1.7	0.24×	0.13 - 0.35
Calcified tissue thickness	mm	0.59	0.47 - 0.72	1.09	0.93 - 1.24	**0.0001**	0.49	0.32 - 0.67	1.94×	1.6 - 2.28
BV/TV	%	25.45	20.77 - 30.12	44.39	38.11 - 50.66	**0.0002**	18.94	11.92 - 25.96	1.83×	1.47 - 2.2
BS/BV	1/mm	15.39	12.91 - 17.86	11.46	9.73 - 13.2	**0.0063**	-3.93	-6.43 - -1.42	0.77×	0.62 - 0.93
BS/TV	1/mm	3.72	3.45 - 3.99	4.91	4.65 - 5.18	**<0.0001**	1.19	0.96 - 1.43	1.33×	1.25 - 1.4
Tb.Pf	1/mm	1.92	0.44 - 3.4	-2.97	-4.19 - -1.77	**0.0001**	-4.89	-6.57 - -3.21	-0.66×	-3.06 - 1.74
SMI		1.37	1.11 - 1.64	0.00	-0.45 - 0.46	**0.0001**	-1.37	-1.86 - -0.87	0.01×	-0.34 - 0.35
Tb.Th	mm	0.26	0.22 - 0.3	0.31	0.28 - 0.35	**0.0247**	0.05	0.01 – 0.09	1.23×	1.06 - 1.41
Tb.N	1/mm	0.97	0.89 - 1.05	1.42	1.33 - 1.51	**<0.0001**	0.45	0.37 - 0.52	1.47×	1.37 - 1.57
Tb.Sp	mm	0.66	0.61 - 0.7	0.52	0.45 - 0.58	**0.0039**	-0.14	-0.2 - -0.08	0.79×	0.7 - 0.87
FD		2.63	2.62 - 2.65	2.66	2.63 - 2.69	**0.019**	0.03	0 - 0.06	1.01×	1 - 1.02
Conn.Dn	1/mm^3	6.20	5.07 - 7.32	8.68	7.05 - 10.31	**0.0029**	2.49	1.1 - 3.87	1.43×	1.19 - 1.68
DA		0.24	0.17 - 0.32	0.34	0.25 - 0.42	0.0589	0.09	0 - 0.19	1.68×	0.87 - 2.49

III. Inter-group comparison with clinical CT

		**mOA**		**sOA**		***P***	**Differences**			
parameter	unit	mean	95% CI	mean	95% CI		Absolute difference	95% CI	Relative (factor)	95% CI
Non-calcified cartilage thickness	mm	2.41	2.04 - 2.77	0.62	0.38 - 0.87	**<0.0001**	-1.78	-2.1 - -1.47	0.26×	0.17 - 0.35
Calcified tissue thickness	mm	0.76	0.6 - 0.93	1.11	1.02 - 1.2	**0.0001**	0.35	0.23 - 0.47	1.54×	1.31 - 1.77
BV/TV	%	18.53	10.43 - 26.64	54.83	39.88 - 69.79	**0.0001**	36.30	23.6 - 49.01	4.16×	1.95 - 6.36
BS/BV	1/mm	15.06	12.74 - 17.38	9.71	7.55 - 11.87	**0.0013**	-5.35	-7.98 - -2.72	0.66×	0.51 - 0.81
BS/TV	1/mm	2.50	1.63 - 3.38	4.78	4.17 - 5.39	**0.0001**	2.28	1.48 - 3.08	2.47×	1.34 - 3.6
Tb.Pf	1/mm	6.74	4.73 - 8.76	1.38	-0.3 - 3.06	**0.0009**	-5.36	-7.87 - -2.86	0.22×	-0.03 - 0.48
SMI		1.58	1.37 - 1.78	0.75	0.4 - 1.1	**0.0009**	-0.83	-1.21 - -0.44	0.48×	0.27 - 0.7
Tb.Th	mm	0.30	0.26 - 0.35	0.44	0.36 - 0.52	**0.0025**	0.14	0.06 - 0.21	1.48×	1.22 - 1.74
Tb.N	1/mm	0.57	0.37 - 0.78	1.22	1.01 - 1.42	**<0.0001**	0.64	0.43 - 0.86	2.75×	1.43 - 4.07
Tb.Sp	mm	0.73	0.6 - 0.85	0.43	0.32 - 0.54	**<0.0001**	-0.30	-0.37 - -0.22	0.59×	0.51 - 0.67
FD		1.89	1.53 - 2.26	2.04	1.7 - 2.39	0.1309	0.15	-0.03 - 0.33	1.10×	0.99 - 1.21
Conn.Dn	1/mm^3	0.96	0.67 - 1.25	1.33	0.91 - 1.74	0.1986	0.37	-0.23 - 0.96	1.93×	0.62 - 3.25
DA		0.90	0.89 - 0.91	0.92	0.9 - 0.93	**0.0234**	0.02	0.01 - 0.03	1.02×	1.01 - 1.03

Table S2. Extended data of Pearson correlation analyses, linear regressions and Bland-Altman plots comparing clinical CT and micro-CT measurements of the ex vivo samples. P, r, r^2 values of correlation analyses, slope and Y-intercept of linear regressions, and bias ± SD of the Bland-Altman plots of Figure 1g, h, l, m, 2b-l, and supplementary Figure S2 are presented. Mild-to-moderate and severe OA groups were pooled (n = 20 or 120 together) for each analysis. Significant (P < 0.05) correlations are indicated with bold numbers.

Parameter	n	*P*	r	r^2	slope	Y-intercept	Bias (Bland-Altman)
Non-calcified cartilage thickness (mean)	20	**6.7×10^{-14}**	0.979	0.958	0.8569	0.07979	-0.16 ± 0.263
Non-calcified cartilage thickness (local)	120	**6.9×10^{-34}**	0.959	0.920	0.8511	0.08857	-0.1608 ± 0.3869
Calcified tissue thickness (mean)	20	**1.8×10^{-4}**	0.743	0.552	0.5894	0.4420	0.0975 ± 0.2132
Calcified tissue thickness (local)	120	**1.7×10^{-10}**	0.712	0.507	0.5905	0.4411	0.09767 ± 0.2685
BV/TV	20	**3.0×10^{-11}**	0.958	0.918	1.933	-30.81	1.767 ± 13.47
BS/BV	20	**2.7×10^{-8}**	0.910	0.828	1.055	-1.775	-1.042 ± 1.714
BS/TV	20	**2.2×10^{-4}**	0.736	0.542	1.607	-3.297	-0.6745 ± 1.14
Tb.Pf	20	**9.5×10^{-10}**	0.939	0.882	1.125	4.655	4.589 ± 1.346
SMI	20	**1.1×10^{-8}**	0.918	0.843	0.6157	0.7379	0.4735 ± 0.4032
Tb.Th	20	**3.4×10^{-6}**	0.841	0.707	1.584	-0.08352	0.0845 ± 0.06962
Tb.N	20	**3.7×10^{-5}**	0.788	0.621	1.330	-0.6914	-0.298 ± 0.2791
Tb.Sp	20	**0.002**	0.643	0.413	1.328	-0.1982	-0.006 ± 0.1719
FD	20	0.557	0.140	0.020	1.891	-3.038	-0.679 ± 0.4877
Conn.Dn	20	0.109	0.369	0.136	0.08380	0.5191	-6.296 ± 2.158
DA	20	0.158	0.328	0.108	0.04421	0.8937	0.616 ± 0.1145

Table S3. Comparison of the relative differences between micro-CT, clinical CT, and MRI. Differences between the gold-standard micro-CT and the lower resolution imaging modalities, reported in previous studies, are expressed in multiplying factors (normalized to micro-CT). Resolution is expressed as width × length × slice thickness. Abbreviations: cCT, clinical CT; µCT, micro-CT; MDCT, multidetector CT; HR-pQCT, high-resolution peripheral quantitative CT; MS-CT, multislice-spiral-CT; SS-CT, singleslice-CT; CBCT, cone-beam CT; Ct.th, calcified tissue-, cortical- or subchondral bone plate thickness

cCT / MRI type, resolution (µm)	µCT resolution (µm)	Ct.th.	BV/TV	BS/BV	BS/TV	SMI	Tb.Th	Tb.N	Tb.Sp	FD	Conn.Dn	DA	Reference
**Comparison of cCT with µCT**													
MDCT<br>287×287×1000	isotropic<br>35 µm	1.18×	0.94×	0.91×	0.82×	0.59×	1.28×	0.72×	0.97×	0.74×	0.16×	4.09×	present study
cCT<br>500 µm slice	50 µm	0.98-0.99×											[30b]
cCT<br>1000 µm slice	50 µm	1.13-1.14×											[30b]
HR-pQCT<br>isotropic 82	isotropic<br>19 µm	1.14×	1.2×	0.75×		0.73×	0.9×	1.2×	0.9×		1×	0.85×	[26e]
MS-CT<br>230×230×500	isotropic<br>20 µm		2.8×				2.4×	1.14×	0.74×				[26a]
SS-CT<br>410×410×1000	isotropic<br>20 µm		5.1×				6×	0.86×	0.66×				[26a]
MDCT<br>350×350×800	isotropic<br>8 µm		1.5×				5.7×	0.2×	4.7×				[26b]
MDCT<br>208×208×500	isotropic<br>16 µm		1.4-2.7×				2.9-4.3×	0.3-0.5×	2.4-8.4×				[26c]
CBCT<br>100	100 µm		1.01×			1.03×	1.02×					0.58×	[28]
MDCT<br>360	100 µm		1.4×			1.04×	2.4×					0.5×	[28]
cCT<br>146×146×600	isotropic<br>50 (45) µm		1.04×				1.85×	0.54×	1.78×		0.16×		[26d]
MS-CT<br>250×250×1000	isotropic<br>30 µm		0.66×				7.3×	0.24×	12.3×				[27e]
**Comparison of MRI with µCT**													
1.5 T<br>195×195×300	isotropic<br>30 µm		1×, 2.5×				2.5×, 1.92×	1.02×, 0.52×	0.79×, 2.91×				[27e]
1.5 T<br>195×195×900	isotropic<br>30 µm		0.58×, 3.33×				3.08×, 2×	1.17×, 0.31×	0.55×, 4.32×				[27e]
1.5 T<br>isotropic 160<br>scaled to 53	isotropic<br>25 µm	1.29×	1.5×	0.62×		1.42×	1.66×	1×	0.92×		0.45×	0.79×	[27g]
3 T<br>230×230×1000	isotropic<br>9 µm		0.07×										[27a]
1.5 T<br>156×156×300	isotropic<br>17.6 µm		3.88×				3.7×	1.04×	0.62×	1.4×			[27b]
1.5 T<br>156×156×500	isotropic<br>26 µm		2.16×				1.63×	1.3×	0.67×				[27c]
3 T<br>156×156×500	isotropic<br>26 µm		3.08×				1.95×	1.55×	0.46×				[27c]
1.5 T<br>156×156×300	isotropic<br>34 µm		2.29×				0.95-1.91×	0.99-1.37×	0.6-0.86×				[27d]
1.5 T<br>156×156×500	isotropic<br>34 µm		2.39×				1.88-2.02×	1.02-1.39×	0.59-0.83×				[27d]
8.5 T<br>isotropic 66	isotropic<br>10 µm		1.66×		1.23×		1.38×		0.74×				[27f]

Table S4. Mean values with their 95% confidence intervals (CI) of the in vivo clinical CT measurements in normal, mild-to-moderate (mOA) and severe OA (sOA) tibial plateaus. n = 9 per group; P values were determined with ANOVA or Kruskal-Wallis ANOVA. Significant ($P < 0.05$) differences are indicated with bold numbers.

		**Normal**		**mOA**		**sOA**		***P***		
parameter	unit	mean	95% CI	mean	95% CI	mean	95% CI	Normal vs. mOA	Normal vs. sOA	mOA vs. sOA
Non-calcified cartilage thickness	mm	2.65	2.32 - 2.98	2.18	2.01 - 2.34	1.30	1.07 - 1.53	**0.0143**	**<0.0001**	**<0.0001**
Calcified tissue thickness	mm	0.85	0.68 - 1.03	0.70	0.63 - 0.77	1.22	1.12 - 1.31	>0.9999	**0.0149**	**0.0005**
BV/TV	%	28.53	20.25 - 36.81	23.23	16.66 - 29.79	50.42	42.07 - 58.78	0.5163	**0.0003**	**<0.0001**
BS/BV	1/mm	6.32	5.11 - 7.52	6.66	5.78 - 7.54	3.58	2.73 - 4.43	>0.9999	**0.0089**	**0.0009**
BS/TV	1/mm	1.67	1.34 - 2	1.52	1.09 - 1.95	1.73	1.49 - 1.97	0.7537	0.9559	0.5797
Tb.Pf	1/mm	-0.34	-1.01 - 0.32	-0.20	-0.97 - 0.57	-0.95	-1.13 - -0.77	0.9173	0.2411	0.1206
SMI		1.29	1 - 1.59	1.34	1.05 - 1.63	0.66	0.51 - 0.82	0.9518	**0.0013**	**0.0006**
Tb.Th	mm	0.78	0.69 - 0.87	0.75	0.66 - 0.84	1.26	1.01 - 1.51	0.954	**0.0002**	**<0.0001**
Tb.N	1/mm	0.36	0.27 - 0.45	0.32	0.21 - 0.42	0.41	0.36 - 0.47	0.6798	0.6279	0.2001
Tb.Sp	mm	1.55	1.28 - 1.81	1.65	1.3 - 2	1.54	1.12 - 1.96	0.8758	0.9993	0.8583
FD		1.85	1.45 - 2.26	2.26	1.63 - 2.89	2.55	1.78 - 3.32	>0.9999	0.5154	>0.9999
Conn.Dn	$1/mm^3$	0.38	0.29 - 0.47	0.35	0.17 - 0.53	0.18	0.06 - 0.29	0.9522	0.0532	0.0972
DA		0.56	0.54 - 0.59	0.52	0.47 - 0.57	0.60	0.54 - 0.66	0.3349	0.3911	**0.0272**

Table S5. Pearson correlation analyses comparing clinical CT and micro-CT measurements of the ex vivo samples to Kellgren-Lawrence (KL) grades and International Cartilage Regeneration and Joint Preservation Society (ICRS) scores. Mild-to-moderate and severe OA groups were pooled (n = 20 together) for each analysis. Significant (P < 0.05) correlations are indicated with bold numbers.

	Micro-CT				Clinical CT			
	KL grade (mean)		ICRS score (mean)		KL grade (mean)		ICRS score (mean)	
	P	r	*P*	r	*P*	r	*P*	r
KL grade (mean)	-	-	**2.1×10^{-6}**	0.850	-	-	**2.1×10^{-6}**	0.850
ICRS score (mean)	**2.1×10^{-6}**	0.850	-	-	**2.1×10^{-6}**	0.850	-	-
Non-calcified cartilage thickness (mean)	**4.2×10^{-6}**	-0.837	**1.7×10^{-4}**	-0.743	**1.4×10^{-6}**	-0.857	**1.9×10^{-4}**	-0.741
Calcified tissue thickness (mean)	**1.1×10^{-4}**	0.759	**0.002**	0.643	**0.006**	0.595	**0.014**	0.539
BV/TV	**1.8×10^{-4}**	0.743	**6.2×10^{-4}**	0.698	**5.5×10^{-4}**	0.702	**3.8×10^{-4}**	0.716
BS/BV	**0.015**	-0.536	**0.018**	-0.521	**0.002**	-0.645	**0.004**	-0.609
BS/TV	**1.2×10^{-6}**	0.859	**4.6×10^{-4}**	0.710	**3.5×10^{-4}**	0.720	**1.5×10^{-4}**	0.747
Tb.Pf	**1.0×10^{-4}**	-0.760	**5.4×10^{-4}**	-0.703	**3.2×10^{-4}**	-0.723	**0.004**	-0.610
SMI	**1.3×10^{-4}**	-0.753	**1.6×10^{-4}**	-0.745	**9.5×10^{-4}**	-0.681	**0.005**	-0.606
Tb.Th	0.088	0.391	0.068	0.416	**0.010**	0.562	**0.006**	0.596
Tb.N	**2.7×10^{-7}**	0.882	**9.6×10^{-5}**	0.762	**2.2×10^{-4}**	0.735	**2.3×10^{-4}**	0.735
Tb.Sp	**0.001**	-0.661	**0.018**	-0.525	**0.001**	-0.668	**1.9×10^{-4}**	-0.740
FD	0.146	0.338	0.242	0.274	0.599	0.125	0.894	0.032
Conn.Dn	0.011	0.556	0.080	0.401	0.135	0.346	0.123	0.356
DA	0.085	0.395	0.238	0.276	0.070	0.414	0.039	0.464

Table S6. Inter-manufacturer and inter-program differences. A summary of studies comparing the relative difference of the results between micro-CT devices of different manufacturers, analyzing the same bone samples at similar resolutions. A summary of studies comparing the relative difference of the results between different analyzing programs, evaluating the same image sets. Differences are expressed in multiplying factors, normalized to the data of an arbitrarily chosen micro-CT device or program described in the studies. Abbreviations: cCT, clinical CT; µCT, micro-CT; HR-pQCT, high-resolution peripheral quantitative CT.

Location,	CT resolution (µm)	BV/TV	BS/BV	SMI	Tb.Th	Tb.N	Tb.Sp	DA	Reference
**µCT vs cCT**									
human tibial plateau	µCT: isotropic 35 µm, cCT: 287×287×1000 µm	0.94×	0.91×	0.59×	1.28×	0.72×	0.97×	4.09×	present study
**Comparison of devices**									
mouse distal femur	3 µCT devices isotropic 7-8 µm	1.27×	1.33×		1.81×	1.9×	1.1×		[29]
mouse distal femur	3 µCT devices isotropic 12-13 µm	1.03×	1.16×		2.17×	2.5×	1.19×		[29]
rat tibia	2 µCT devices isotropic 11-12 µm	1.08×	1.03×	1.06×	0.77×	1.21×	0.99×		[39]
**Comparison of programs**									
human trapezium, radius	µCT 20 µm, same image set with 2 programs	0.99×	1.24×		1.15×	0.58×	1.06×		[40]
human trapezium, radius	HR-pQCT 60.7 µm, same image set with 2 programs	0.98×			1.19×	0.52×	1.13×		[40]
human radius, spine, femur	µCT 18 µm, same image set with 4 programs	1×			0.9×		0.88×	1.81×	[41]

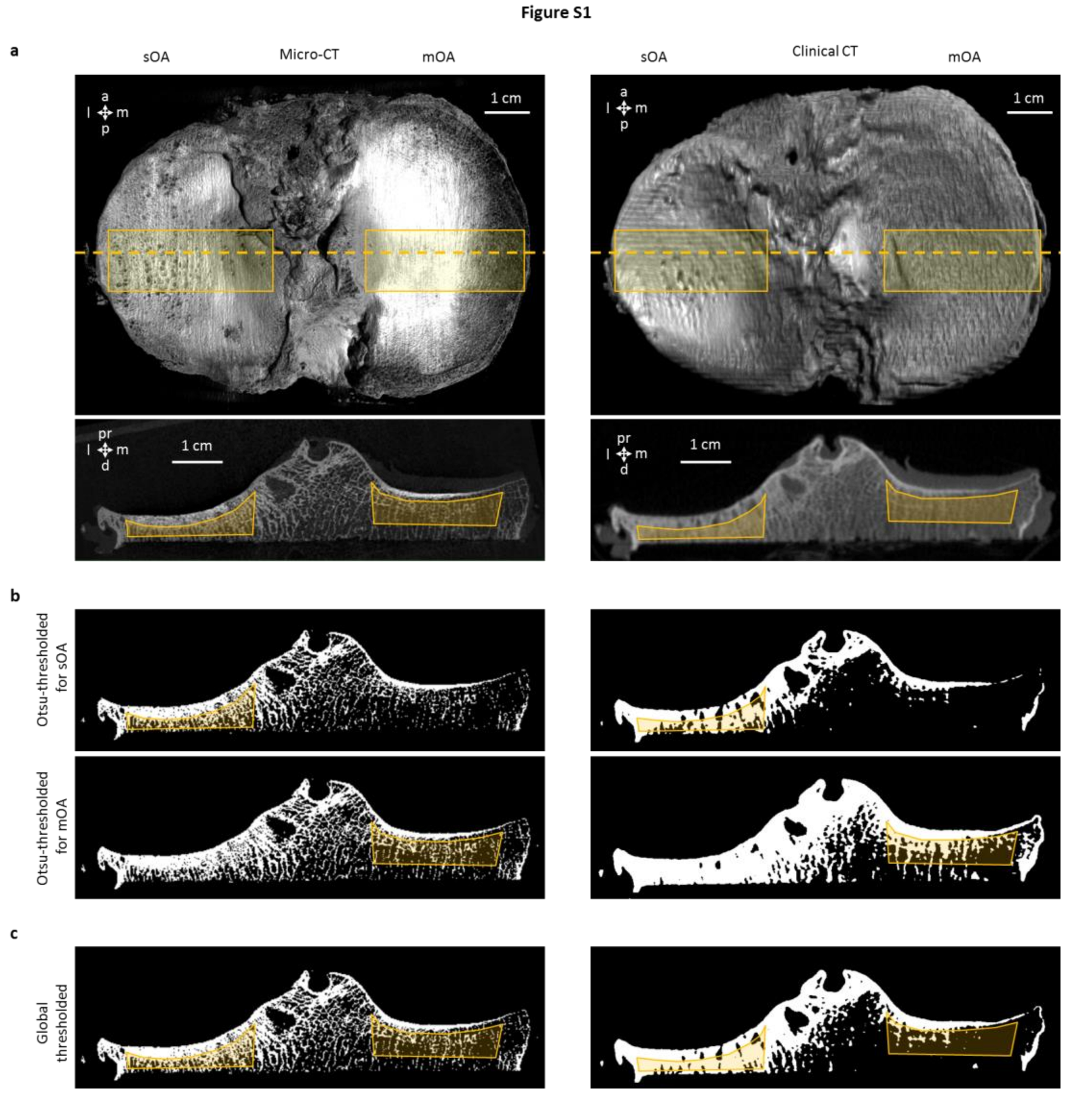

Figure S1. Overview of the structural analysis protocol. **(a)** Representative 3D reconstructed CT models and 2D sections, acquired with micro-CT and clinical CT of the same sample, showing the position of the evaluated volumes of interests (VOI) within the subarticular spongiosa. VOIs of the severe OA (sOA) and mild-to-moderate OA (mOA) tibial plateaus were selected manually. Dashed line indicates the location of the 2D section. **(b)** Binarized images of the subchondral bone with Otsu's automatic method, resulting in different threshold values for each VOI. **(c)** Binarized images of the subchondral bone, using the mean of the threshold values acquired with Otsu's method, as a second, global thresholding of the VOIs for subsequent determination of 3-dimensional bone microstructural parameters. Abbreviations: a, anterior; d, distal; l, lateral; m, medial; p, posterior; pr, proximal.

Figure S2

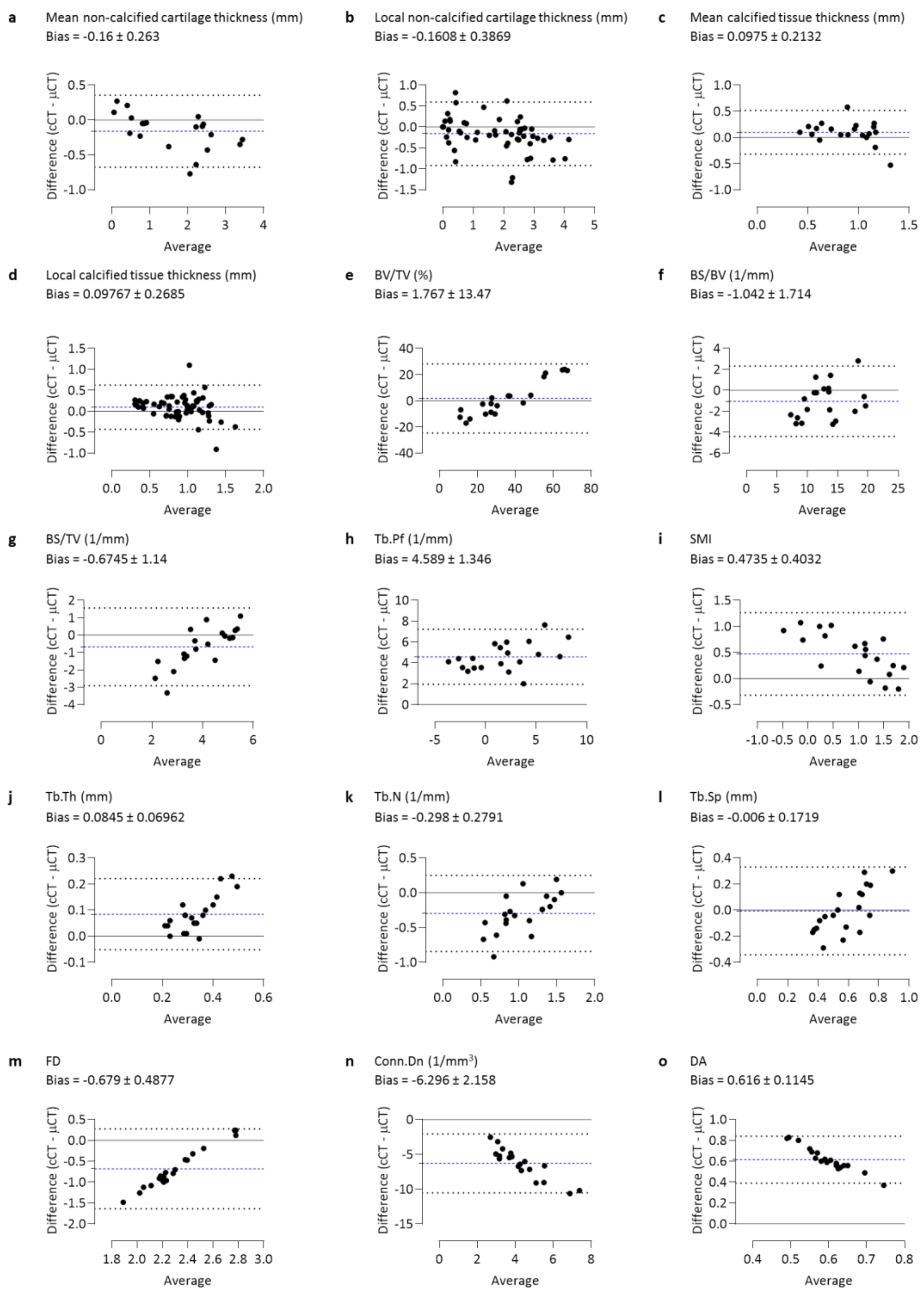

Figure S2. Bland-Altman plots for the difference between clinical CT (cCT) and micro-CT (µCT) measurements. Bland-Altman plots of the **(a)** mean and **(b)** local non-calcified cartilage thickness, **(c)** mean and **(d)** local calcified tissue thickness, and **(e)** percent bone volume (BV/TV), **(f)** bone surface-to-volume ratio (BS/BV), **(d)** bone surface density (BS/TV), **(e)** trabecular pattern factor (Tb.Pf), **(f)** structure model index (SMI), **(g)** trabecular thickness (Tb.Th), **(h)** trabecular number (Tb.N), **(i)** trabecular separation (Tb.Sp), **(j)** fractal

dimension (FD), **(k)** connectivity density (Conn.Dn), and **(l)** degree of anisotropy (DA) of the subarticular spongiosa. n = 20 or 120 per group (mOA and sOA pooled together). Black dotted lines: limits of agreement, blue dashed line: bias.

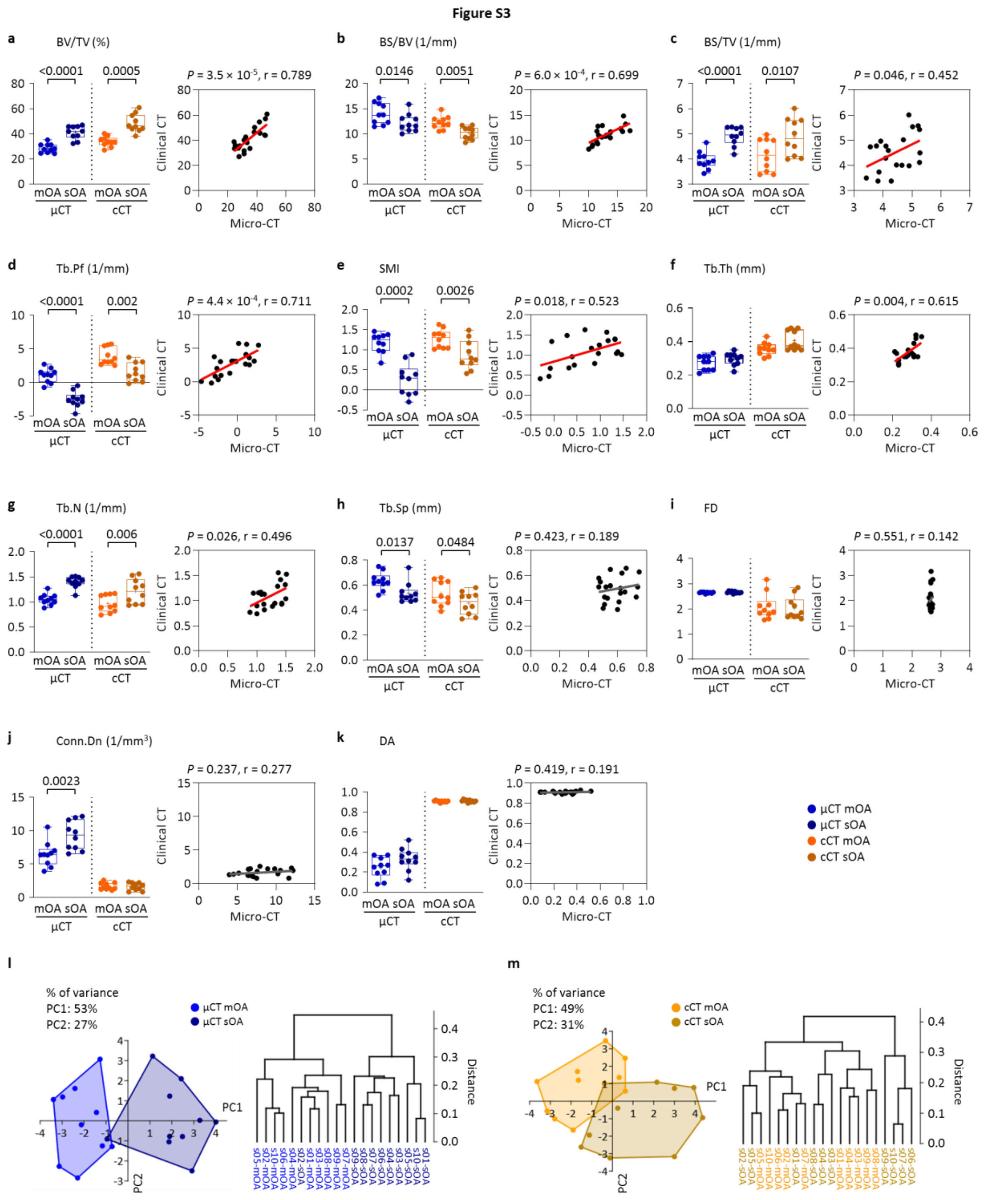

Figure S3. Comparison of the subarticular spongiosa microstructure of mild-to-moderate and severe OA tibial plateaus with clinical CT and micro-CT, thresholded with Otsu's method.[35] Box plots of the measured values, and scatter plot and linear regression showing the correlation between clinical CT and micro-CT measurements of the **(a)** percent bone volume (BV/TV), **(b)** bone surface-to-volume ratio (BS/BV), **(c)** bone surface density (BS/TV), **(d)** trabecular pattern factor (Tb.Pf), **(e)** structure model index (SMI), **(f)** trabecular thickness (Tb.Th), **(g)** trabecular number (Tb.N), **(h)** trabecular separation (Tb.Sp), **(i)** fractal dimension (FD), **(j)** connectivity density (Conn.Dn), and **(k)** degree of anisotropy (DA). *n* = 10 per

group; paired T-test or Wilcoxon test. Principal components analysis and cluster analysis based on the above subarticular spongiosa parameters determined with **(l)** micro-CT or **(m)** clinical CT. Data points represent individual mild-to-moderate or severe OA tibial plateaus (*n* = 10 per group). Information content (% of variance) of axes for principal components 1 (PC1) and 2 (PC2) are shown above graphs. Abbreviations: mOA, mild-to-moderate OA; sOA, severe OA; cCT, clinical CT; μCT, micro-CT.

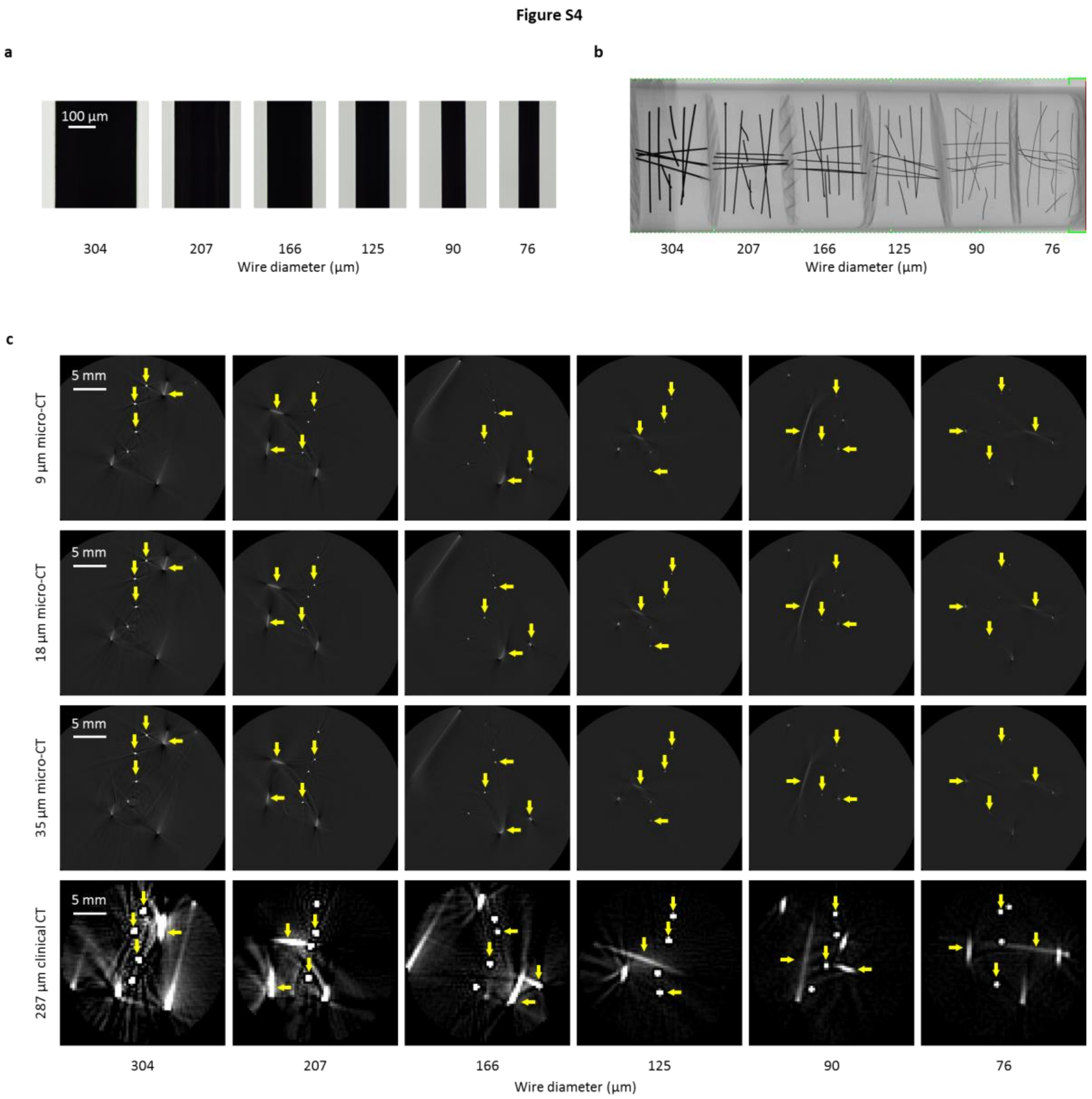

Figure S4. Assessment of the visibility of thin structures with a custom made phantom. Tungsten wires with 76, 89, 126, 167, 207 and 307 µm nominal diameters (Needle cleaning kit, Sigma-Aldrich, Taufkirchen, Germany) were cut into 12-12 pieces, **(a)** each were measured at 3-3 locations under an Olympus BX45 microscope equipped with a 20× objective with the CellSens software (Olympus, version 1.12) (actual mean diameters: 76, 90, 125, 166, 207 and 304 µm). The wires were manually inserted into a polyester sponge approximately perpendicular to each other (4-4 pieces in 3 planes: 1. horizontal, parallel with the slicing plane; 2. vertical, parallel with the slicing plane; and 3. horizontal, perpendicular to the slicing plane of the CT scans), and scanned with micro-CT with 9, 18, and 35 µm isotropic resolutions and with clinical CT with 287×287×1000 µm resolution. **(b)** Micro-CT overview of the phantom. **(c)** Representative images of the different resolution scans of the different

diameter wires (arrows). Note, the presence of metal artifacts, especially at larger diameters and in the clinical CT scans, limits the accuracy of thickness measurements.

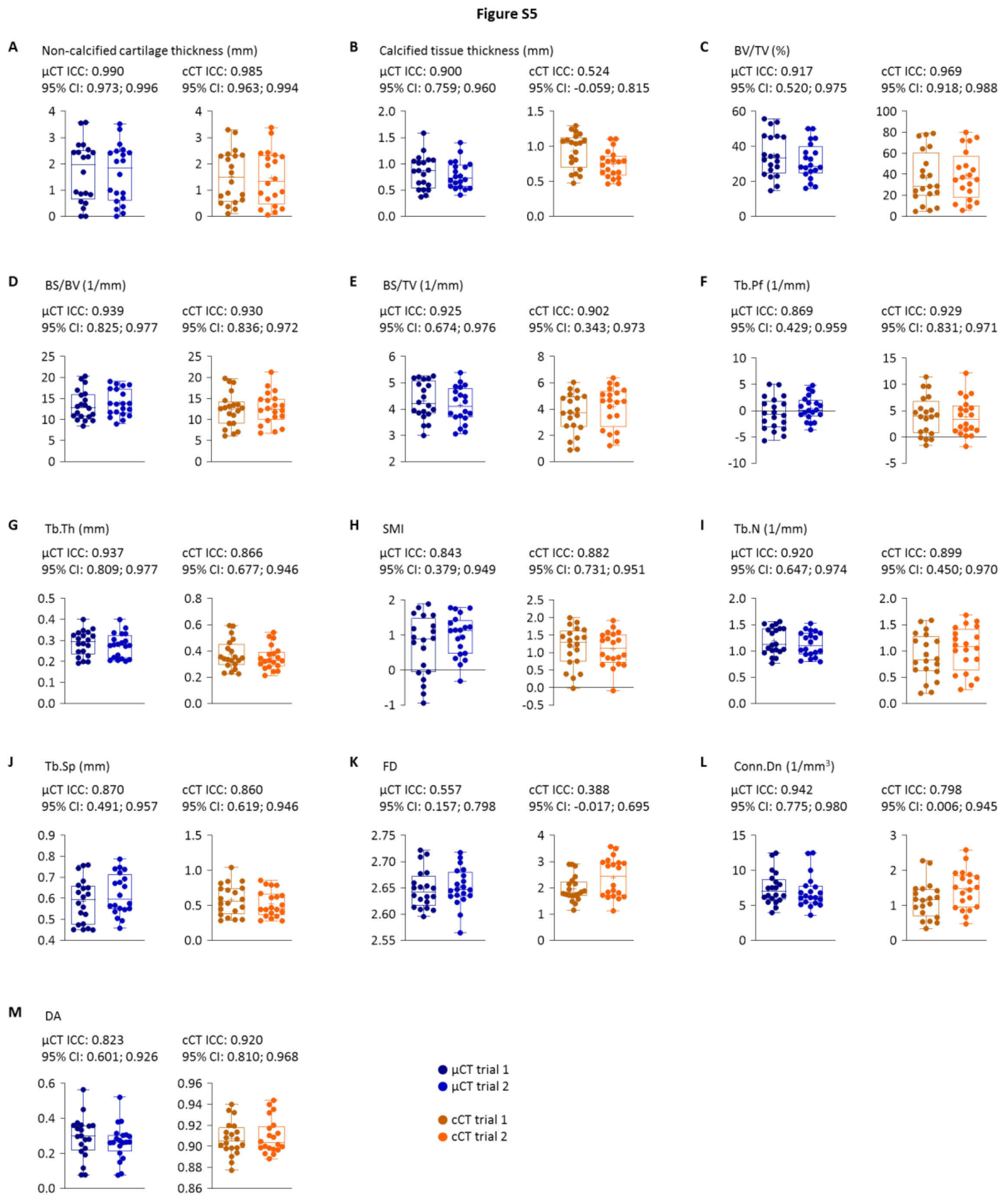

Figure S5. Intraobserver comparison of the measurements. Non-calcified cartilage and calcified tissue thicknesses were measured manually, and VOIs of the subarticular spongiosa were selected manually twice with 3.5 months difference by the same observer, and were evaluated automatically. Intraclass correlation coefficients (ICC) were calculated with "absolute agreement", and the results are shown above the graphs together with their 95% confidence intervals (CI). Box plots of the **(a)** non-calcified cartilage thickness, **(b)** calcified tissue thickness, **(c)** BV/TV, **(d)** BS/BV, **(e)** BS/TV, **(f)** Tb.Pf, **(g)** Tb.Th, **(h)** SMI, **(i)** Tb.N, **(j)** Tb.Sp, **(k)** FD, **(l)** Conn.Dn, and **(m)** DA.

Figure S6

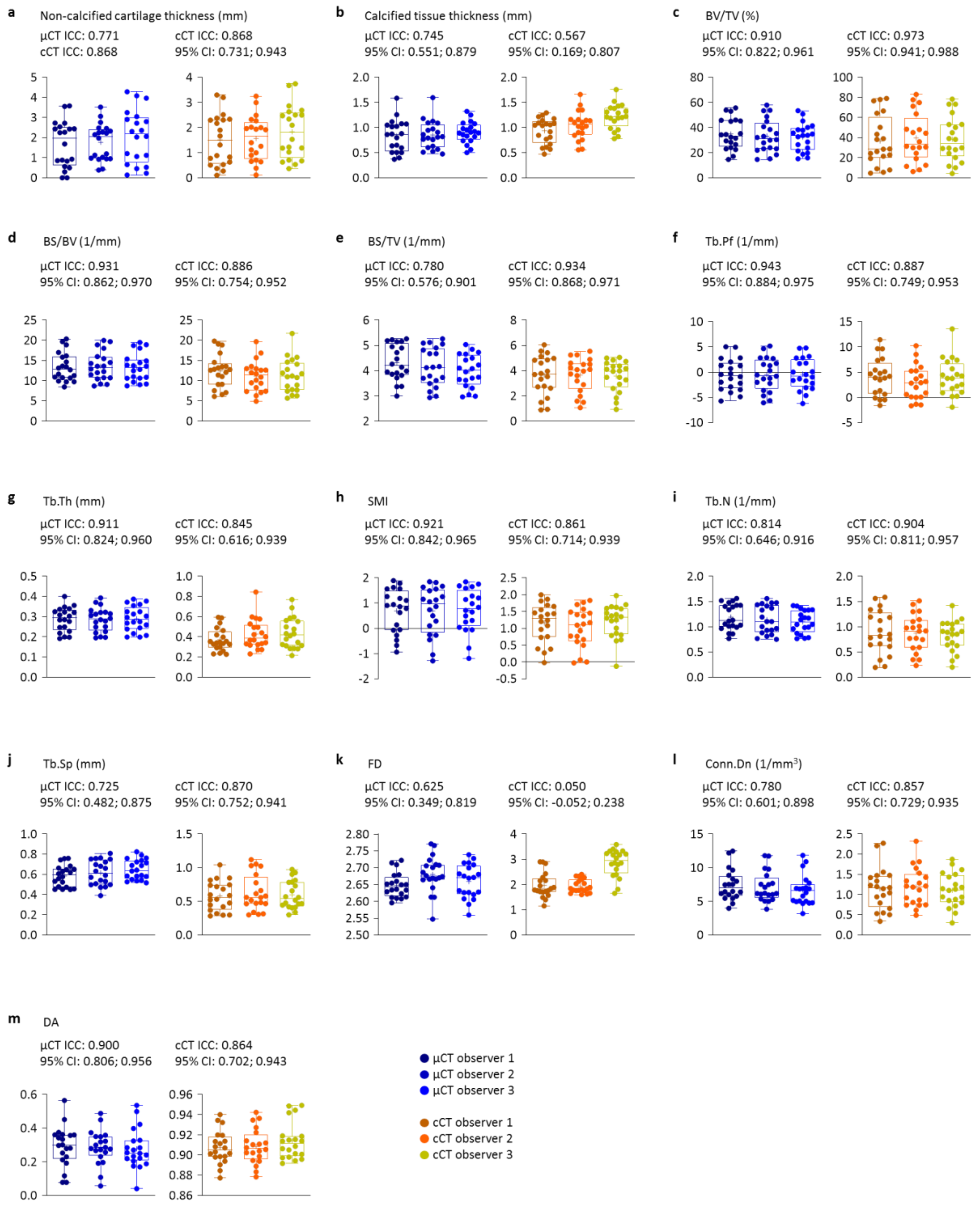

Figure S6. Interobserver comparison of the measurements. Non-calcified cartilage and calcified tissue thicknesses were measured manually, and VOIs of the subarticular spongiosa were selected manually by 3 independent observers, blinded to each other, and were evaluated automatically. Intraclass correlation coefficients (ICC) were calculated with “absolute agreement”, and the results are shown above the graphs together with their 95% confidence intervals (CI). Box plots of the **(a)** non-calcified cartilage thickness, **(b)** calcified tissue

thickness, **(c)** BV/TV, **(d)** BS/BV, **(e)** BS/TV, **(f)** Tb.Pf, **(g)** Tb.Th, **(h)** SMI, **(i)** Tb.N, **(j)** Tb.Sp, **(k)** FD, **(l)** Conn.Dn, and **(m)** DA.

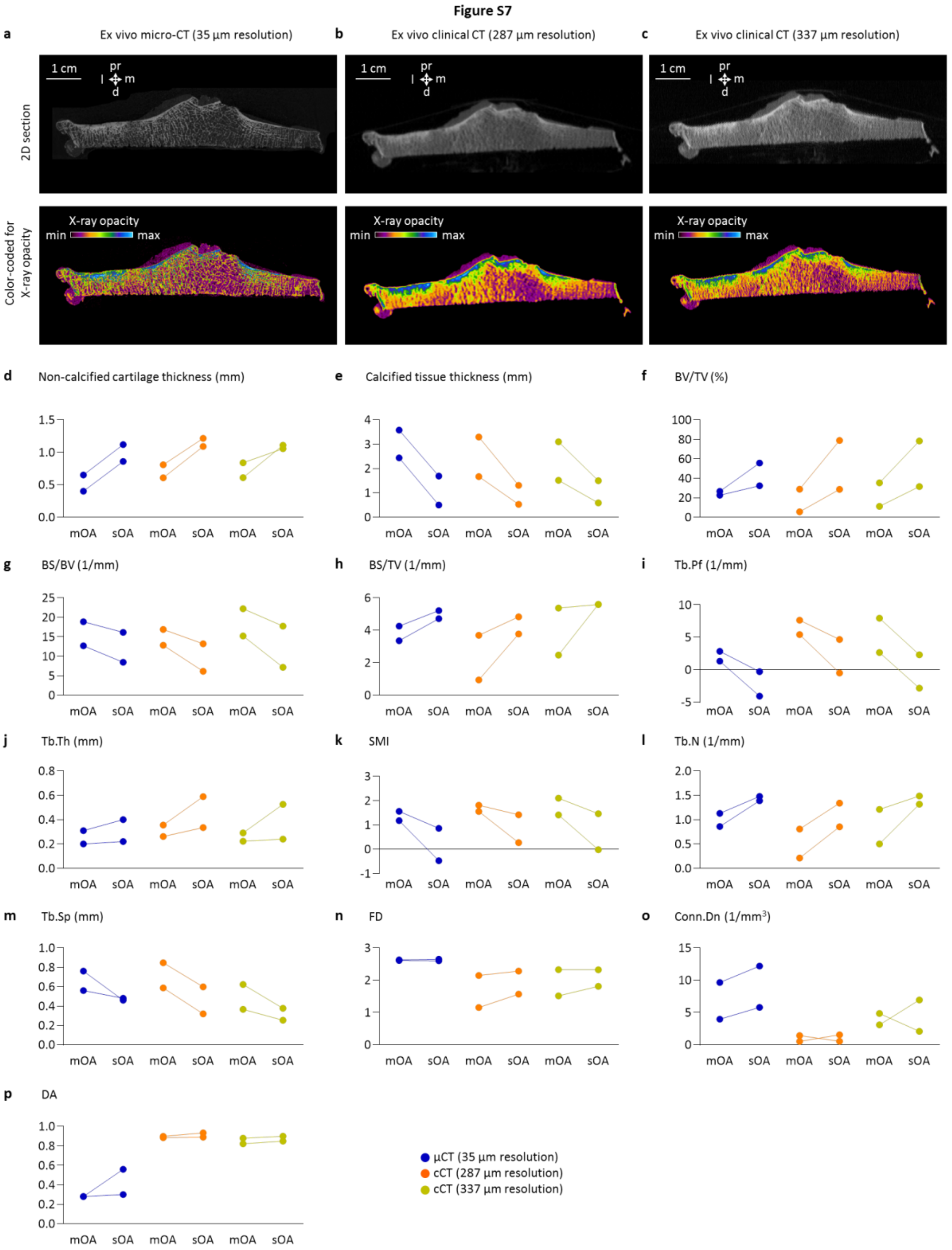

Figure S7. Comparison of different resolution ex vivo CT scans. Representative 2D grayscale sections, and ones color-coded for X-ray opacity of an ex vivo human proximal tibia sample, scanned with **(a)** micro-CT at 35 µm resolution, **(b)** clinical CT at 287 µm resolution, and **(c)** the same clinical CT dataset re-reconstructed with a lower spatial resolution of 337 µm, similar to that of in vivo scans. Two pairs of representative mild-to-moderate OA (mOA) and severe OA (sOA) (2 medial and 2 lateral tibial plateaus of 2 proximal tibiae) samples were

compared quantitatively regarding **(d)** non-calcified cartilage thickness, **(e)** calcified tissue thickness, **(f)** BV/TV, **(g)** BS/BV, **(h)** BS/TV, **(i)** Tb.Pf, **(j)** Tb.Th, **(k)** SMI, **(l)** Tb.N, **(m)** Tb.Sp, **(n)** FD, **(o)** Conn.Dn, and **(p)** DA. For better visual comparability, a low number of specimens (*n* = 2 per group) was used. Statistical comparison was not performed.

Figure S8

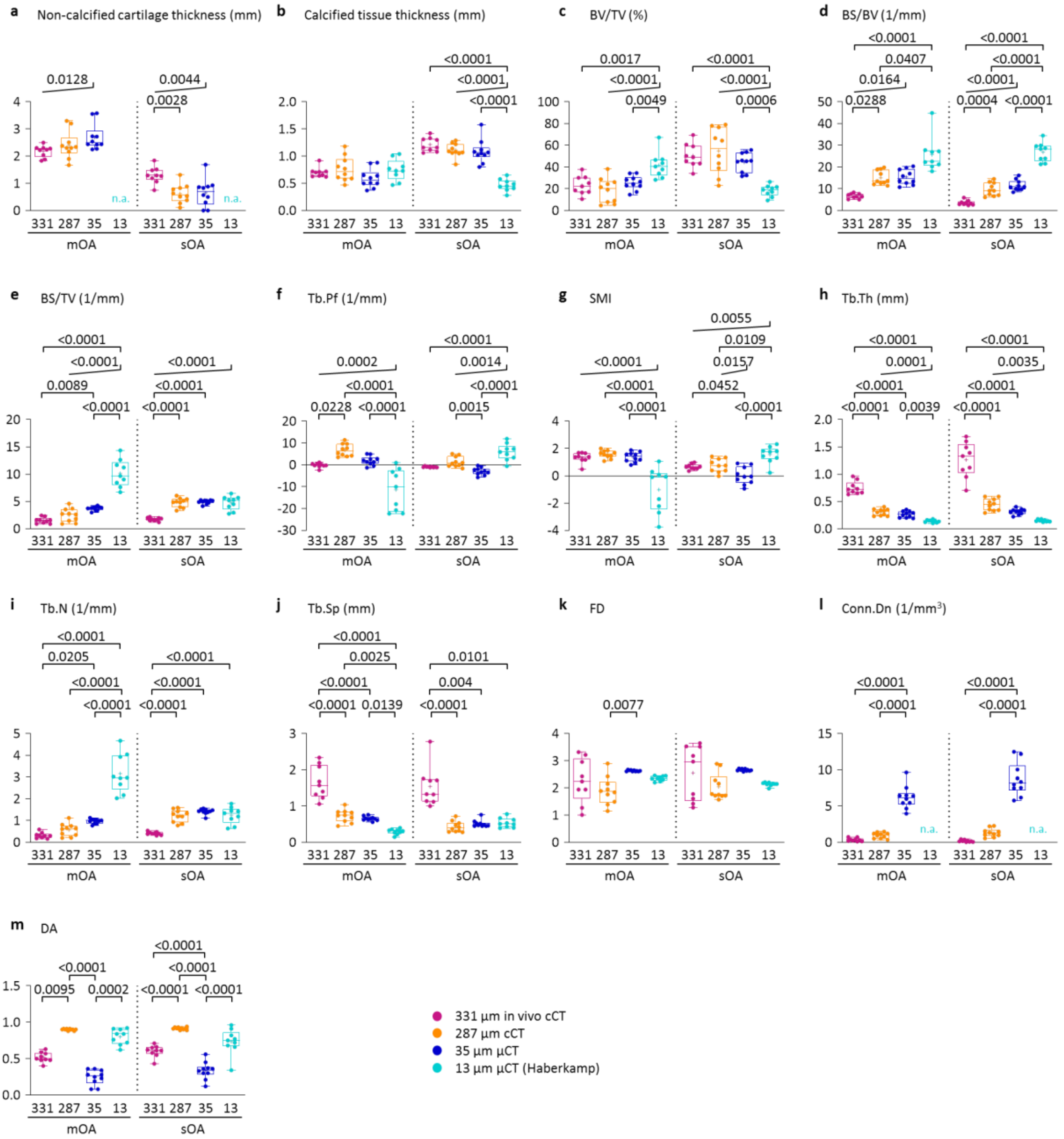

Figure S8. Side by side comparison of 331, 287, 35 and 13 µm resolution CT techniques. Box plots of the **(a)** non-calcified cartilage thickness, **(b)** calcified tissue thickness, **(c)** BV/TV, **(d)** BS/BV, **(e)** BS/TV, **(f)** Tb.Pf, **(g)** SMI, **(h)** Tb.Th, **(i)** Tb.N, **(j)** Tb.Sp, **(k)** FD, **(l)** Conn.Dn, and **(m)** DA. Two sets of samples of the present study [(i) patient knees scanned in vivo at 331 µm resolution, and (ii) tibial plateaus scanned ex vivo at 287 and 35 µm], were compared to a third set from a previous study [(iii) tibial plateau samples scanned ex vivo at 13 µm (Haberkamp et al. 2020)[2e]] with ANOVA or Kruskal-Wallis ANOVA. Note, missing data of non-calcified cartilage thickness and Conn.Dn of the Haberkamp study was denoted as n.a. on the graphs. n = 9 or 10 per groups. Numbers under the graphs indicate the resolution

(in µm) used for data acquisition. Abbreviations: mOA, mild-to-moderate OA; sOA, severe OA; cCT, clinical CT; µCT, micro-CT.

Figure S9

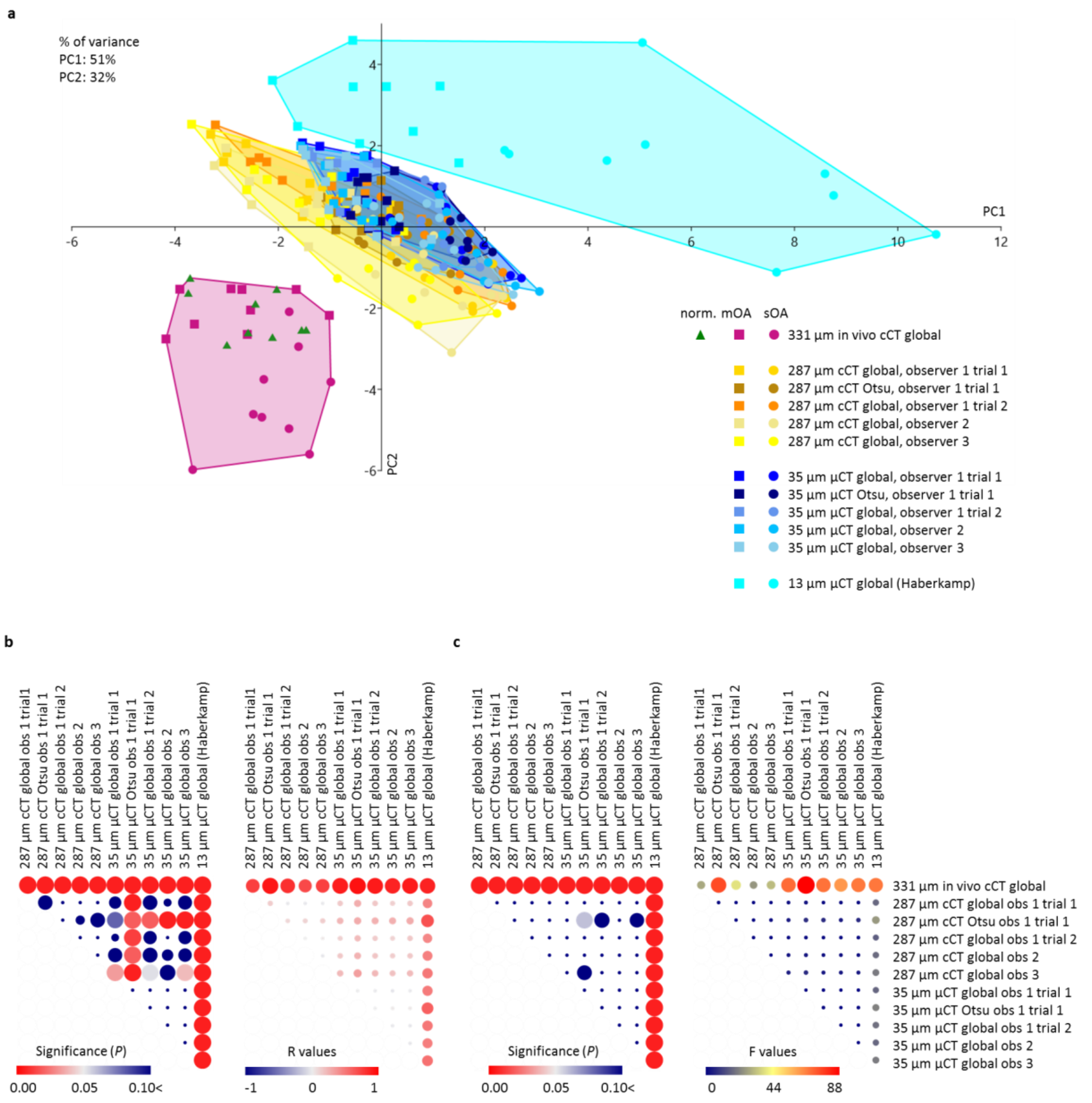

Figure S9. Multivariate comparison of 331, 287, 35 and 13 µm resolution CT techniques, considering inter/intraobserver differences. Raw data (BV/TV, BS/BV, BS/TV, Tb.Th, Tb.Sp, Tb.N, Tb.Pf, and SMI) from a previous study (Haberkamp et al. 2020[2e] at 13 µm) were re-evaluated together with the present study's data including data of intra- (trial 1-2) and interobserver (observer 1-3) measurements, global and Otsu's thresholding with **(a)** principal components analysis, **(b)** one-way analysis of similarities (ANOSIM) and **(c)** one-way permutational multivariate analysis of variance (PERMANOVA), to compare the similarity of the datasets. 'Unreliable' parameters (calcified tissue thickness, FD, Conn.Dn, and DA) were excluded from the analysis. Larger dots indicate lower (more significant) *P*, and higher R and F values (greater difference between the groups). Abbreviations: norm., normal; mOA, mild-to-moderate OA; sOA, severe OA; cCT, clinical CT; µCT, micro-CT; obs, observer.

Figure S10

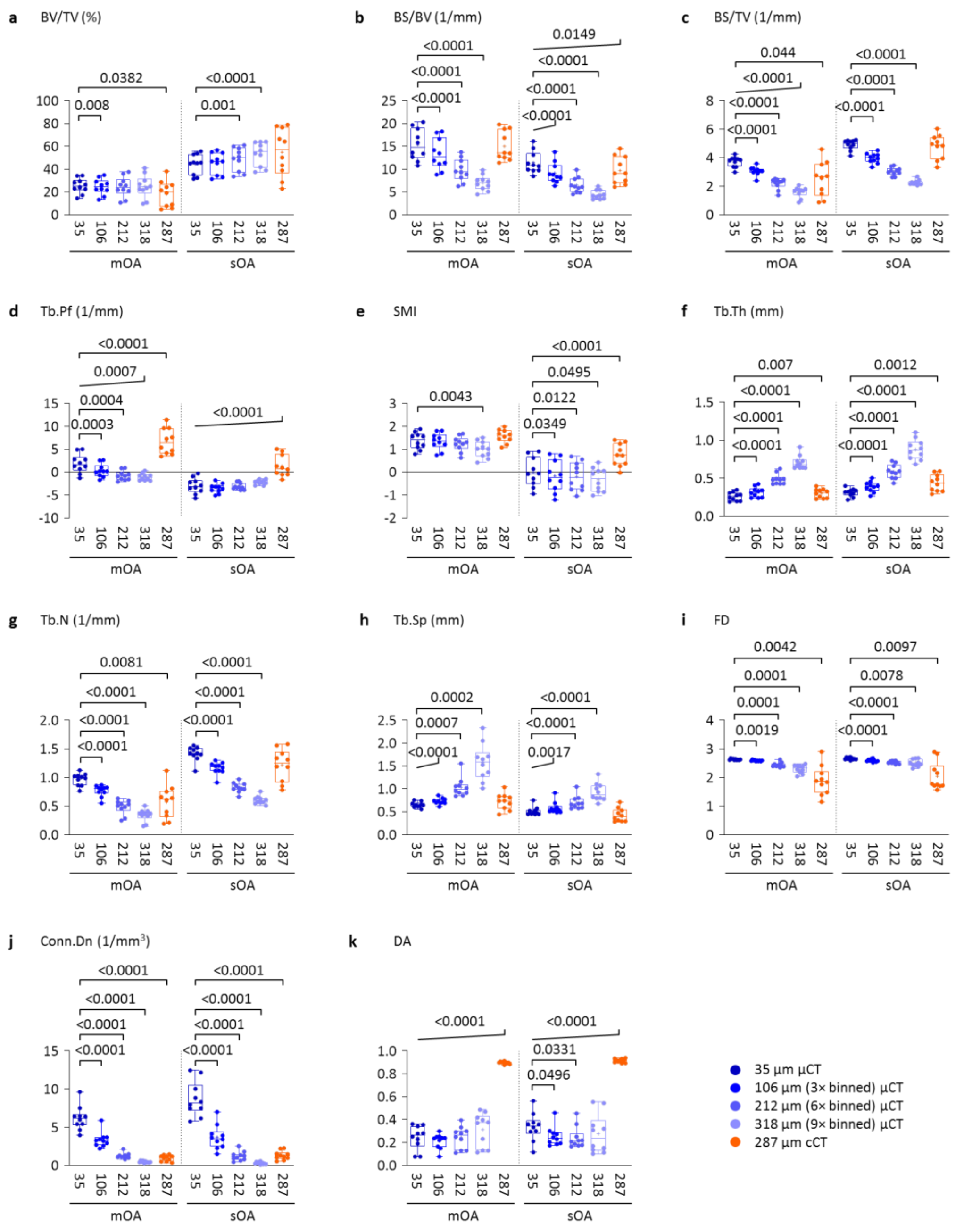

Figure S10. Comparison of the subarticular spongiosa microstructure of mild-to-moderate and severe OA tibial plateaus with clinical CT and micro-CT with 3D step-wise binning to 106, 212, and 318 µm nominal isotropic resolutions. Box plots of the measured values of the **(a)** percent bone volume (BV/TV), **(b)** bone surface-to-volume ratio (BS/BV), **(c)** bone surface density (BS/TV), **(d)** trabecular pattern factor (Tb.Pf), **(e)** structure model index (SMI), **(f)** trabecular thickness (Tb.Th), **(g)** trabecular number (Tb.N), **(h)** trabecular separation (Tb.Sp), **(i)** fractal dimension (FD), **(j)** connectivity density (Conn.Dn), and **(k)** degree of anisotropy (DA). *n* = 10 per group; all groups were compared to 35 µm micro-CT

with repeated measures ANOVA and Dunnet's test. Abbreviations: mOA, mild-to-moderate OA; sOA, severe OA; cCT, clinical CT; μCT, micro-CT.

Supplementary Data 1. Raw data of the figures. Excel file containing the raw data of the box plots, multivariate analyses, and correlation analyses shown in the figures and tables of the manuscript. For detailed description please refer to the figure and table legends.
